# Management and Prevention of Neurodegenerative Disorders: Can Antioxidant-Rich Dietary Interventions Help?

**DOI:** 10.3390/antiox14091078

**Published:** 2025-09-02

**Authors:** Diksha Nagpal, Shivangi Nema, Shakti Nagpal, Murali Monohar Pandey, Deepak Kaushik, Himanshu Kathuria

**Affiliations:** 1 Department of Pharmaceutical Sciences, Maharshi Dayanand University, Rohtak 124001, Haryana, India; diksha07nagpal@gmail.com (D.N.); deepkaushik1977@gmail.com (D.K.); 2 Department of Pharmacy, Birla Institute of Technology and Science, Vidya Vihar Campus, Pilani 333031, Rajasthan, India; p20220065@pilani.bits-pilani.ac.in; 3 Department of Pharmacy, National University of Singapore, Singapore 117559, Singapore; shakti.nagpal@u.nus.edu; 4 Nusmetics Pte Ltd., E-Centre@Redhill, Singapore 139471, Singapore

**Keywords:** neurodegenerative disease, clinical status, diet interventions, pharmacological role, antioxidants, reactive oxygen species

## Abstract

Neurodegenerative diseases are associated with the senescence of functional neurons, which hampers brain functions. These diseases are caused by the accumulation of reactive oxygen species, reactive nitrogen species, cholinesterase malfunction, neuronal inflammation, and mitochondrial dysfunction. The incidence of neurodegenerative disease has been on the rise. Current therapeutic interventions are expensive, exhibit poor efficacy, and have numerous side effects. Several studies have explored the potential of crucial dietary substances rich in antioxidants and micronutrients in alleviating the clinical manifestations of such deadly diseases. Consumption of sufficient antioxidants, fatty acids, and polyphenols in regular diets delays the onset of neurodegenerative diseases. Several medicinal plants, such as Withania somnifera, *Curcuma longa*, Panax ginseng, Ginkgo biloba, aloe vera, Punica granatum, and various phytoextracts, contain such micronutrients in reasonable amounts. Specific dietary interventions, supplements, and patterns such as the Mediterranean-DASH intervention for neurodegenerative delay, ketogenic, paleolithic, and Wahls elimination diets have been beneficial in neurodegenerative conditions. These diet interventions and other functional foods can be an attractive, non-invasive, and inexpensive approach in the management and prevention of neurodegenerative conditions. This review discusses potential pharmacological bases involved in neurodegeneration, covering mitochondrial damage, impaired mitophagy, neuroinflammation, ferroptosis, glymphatic clearance dysfunction, brain–body interactions, and disruption of vagus nerve stimulation. The review further highlights clinical diet interventions and assorted functional foods, including fruits, vegetables, vitamins, specific supplements, and special diets, for neurodegenerative conditions. The discussion extends insights into clinical research and trials of these functional foods under neurodegenerative conditions. Overall, dietary interventions show promise in the prevention and management of neurodegenerative conditions.

## 1. Introduction

Neurodegenerative disorders are a group of heterogeneous disorders resulting from the progressive loss of neurons [1,2]. It is a major public health concern worldwide, which is also a financial and social burden on the diseased family [3]. The increasing prevalence of these diseases is strongly linked to the growing elderly population [4]. With an expanding elderly population, the financial burden of age-related health disorders is increasing, especially in low- and middle-income countries. These disorders are the second leading cause of death globally, accounting for approximately 9 million deaths per year [5]. As per a WHO report, the major contributors to neurological Disability-Adjusted Life Years (DALYSs) were around 42.2% stroke, 16.3% migraine, 10.4% dementia, 7.9% meningitis, and 4.9% epilepsy [5]. As per WHO report, around 55 million people worldwide currently suffer from dementia, which is projected to rise to 78 million by 2030 and 139 million by 2050 [6]. Neurodegenerative diseases mainly include Alzheimer’s disease (AD) [7], multiple sclerosis (MS) [8], Parkinson’s disease (PD) [9], Huntington’s disease (HD) [10], Amyotrophic lateral sclerosis (ALS) [11], and Lewy body diseases [12]. These diseases are pathologically described as a dysfunction or progressive loss of functional neurons [13]. According to the reports in 2023, in America, around 6.7 million people aged 65 and older have AD. This number is expected to grow to 13.8 million by 2060 [14].

The top contributing factors include age-related, continuous mutations in mitochondrial DNA, which can result in mitochondrial dysfunction, oxidative stress, as well as altered metabolism [15,16]. Aging leads to a reduction in brain size and weight, which is related to reduced total cerebral, gray, white matter, and hippocampal volumes [17]. However, a thorough understanding of disease progression is still evolving. Furthermore, effective preventative and therapeutic interventions are also needed to manage the population’s health [15,16]. Another pathological reason is protein misfolding; for example, alteration in amyloid-β and tau proteins progresses to AD. These misfolded proteins activate the NF-kB pathway, which upregulates TNF-α and interleukins-1β [18,19]. This results in activation of COX-2, inducing nitric oxide synthase, dysregulating GSK3β signaling, and leading to hyperphosphorylation of such proteins [20,21,22]. Other than these, the OXR1 (Oxidative Resistance 1) genetic variant has been associated with cerebellar atrophy, language delay, hypotonia, and seizures. Overexpression of OXR1 improves survival in the ALS mouse model and imparts protection against neurodegenerative diseases [23]. Further, oxidative stress caused by imbalance and overproduction of reactive oxygen species (ROS) and reactive nitrogen species (RNS) damages cellular biomolecules, causing neural damage leading to neurodegenerative symptoms [24]. Free radicals such as hydroxyl radicals, superoxide, and singlet oxygen can attack healthy cells and cause structural deformities in proteins [25]. Antioxidants can deactivate and stabilize these free radicals before they attack the cells.

Among all neurodegenerative diseases, AD is the most common, which causes an amnestic cognitive impairment and is characterized by the presence of β-amyloid plaques and tau neurofibrillary tangles [26]. Patients with PD show impaired cognitive skills, memory, speech, judgment, and other psychiatric disturbances [27]. PD symptoms include tremors, muscle rigidity, speech problems, and difficulty maintaining posture. This mainly results from the loss of dopaminergic neurons in the substantia nigra pars compacta [28]. On the other hand, multiple sclerosis is caused by genetic predisposition and other environmental factors, such as low sun exposure, low vitamin D levels [29], viral exposure, and smoking [30]. The alterations in diet have been shown to have preventative effects on such neurodegenerative diseases [31,32]. The unhealthy lifestyle choices increase the risk of such chronic illnesses [3].

Since ancient times, natural products have been used in the prevention and treatment of these ailments [33]. Polyphenols, including flavonoids, stilbenes, curcuminoids, and phenolic acids, are commonly found in herbs, tea, seeds, fruits, cereals, and red wine and show underlying health benefits [34,35]. Foods rich in antioxidants, like fruits, vegetables, seeds, and whole grains, have been shown to help fight neurodegenerative diseases [36]. Such foods, which offer health benefits beyond their basic nutritional value, are known as functional foods [37]. They are often consumed regularly and are fortified with essential vitamins, proteins, fats, and powerful antioxidants [37,38]. For example, vitamin D is often fortified in milk, which modulates neuron production, neurotrophic factors, nitric oxide synthase, and glial cell-derived neurotrophic factor, providing neuroprotective actions [39]. Likewise, fatty acids, such as omega-3 fatty acids and polyunsaturated fatty acids (PUFAs), have been known to modulate the risk of developing cognitive disability and dementia [40]. Similarly, docosahexaenoic acid (DHA) plays a role in maintaining neuronal function and modifying the expression of genes essential for cognitive health [41,42]. PUFAs reduce inflammation by inhibiting the production of inflammatory cytokines. Also, the beneficial effects of PUFAs in inducing the PPAR (peroxisome proliferator-activated receptors) have been described [43,44]. Other herbs such as black cumin, ginkgo, and garlic have been shown to prevent neurodegeneration through an antioxidant mechanism [45]. Recently, mushrooms have shown antioxidant, anti-inflammatory, antivirus, immune-modulating, and anti-diabetic activities [46]. Another popular food is green tea infusion, which contains polyphenolic compounds such as epigallocatechin-3-gallate, epicatechin gallate, epigallocatechin, and epicatechin, the primary polyphenol that shows multiple health benefits [47,48]. Dietary molecules like α-lipoic acid and polyphenols inhibit the NF-kβ pathways, resulting in reduced inflammation [49]. Specifically, epigallocatechin-3-gallate has shown suppressive effects in Huntington’s disease model [50]. In another study, omega-3 fatty acid (DHA and eicosapentaenoic acid) supplementation improved cognitive performance [51,52]. Probiotics are also becoming popular for their effectiveness in neurodegenerative diseases. Various clinical trials are studying the consumption of probiotics for PD (NCT04451096, NCT03968133, and NCT05173701).

Current therapeutic interventions alleviate symptoms but cannot arrest the development of neurodegeneration [43,53,54]. Other than this, functional foods and dietary interventions play an essential role in managing various chronic diseases [55]. Across the globe, one out of every five deaths occurs due to the consumption of a suboptimal diet, and those having metabolic disorders (e.g., obesity and diabetes) are at greater risk of developing dementia, cognitive defects, and Alzheimer’s disease [56]. Modern pattern of nourishment, including Western diets, uses ultra-processed foods with simple and refined carbohydrates, refined substances, fats, and cholesterol [57]. Specifically, the Western diet has been associated with a high risk of dementia, enhanced production of β-amyloid protein, and sped-up inflammation in the brain [58,59]. Appetitive peptides like insulin, leptin, and orexin are known to regulate food consumption and body weight [60]. They are involved in the modulation of neuroendocrine, cognitive, and immune functions. For example, insulin and leptin act on the hippocampus to promote hippocampal synaptic plasticity, increase neurogenesis, and enhance synaptic transmission. Diets influencing these peptides can modulate brain functions. Western diets also affect gut functioning, gut microbiota composition, and reduce nutrient absorption [61]. This alteration of gut microbiota composition has been linked to obesity, the onset of type 2 diabetes, and metabolic syndrome [62].

Approximately 40% of cases of dementia can be prevented by modifying lifestyle and dietary patterns. Various initiatives and programs have been developed with focus on ‘Food as Medicine’. These efforts integrate nutrition with healthcare, emphasizing the vital role that balanced, healthful eating plays in overall well-being. These initiatives harness the power of food to prevent, manage, and even treat various health conditions in a holistic approach with medical care [63]. These initiatives include medically customized meals (therapeutic meals) to fit a person’s specific needs. These medically tailored meals, groceries, and produce prescriptions have been shown to improve diabetes, chronic liver disease, and heart failure. A cohort study involved 1020 participants receiving medically tailored meals [64]. Results indicated a 16% reduction in overall healthcare costs, 49% fewer hospital admissions, and 72% fewer admissions to nursing facilities. Medically tailored meals are necessary for patients with complex medical conditions who are unable to prepare or shop for meals. Medically tailored groceries are applicable for a broader range of patients suffering from acute and chronic conditions who can still prepare food at home. While ‘produce prescriptions’ are applicable for a much broader set of recipients for disease prevention and management [65]. Several clinical studies have explored the use of food as a form of medicine for diabetes. A randomized clinical trial (NCT04828785) on 200 participants tested the efficacy of medically tailored meals in patients with Type 2 Diabetes Mellitus.

This review discusses the pharmacological considerations and mechanism of neurodegeneration, including mitochondrial damage, neuroinflammation, glymphatic clearance, ferroptosis, brain–body interactions, and vagus nerve stimulation. It focuses on the role of specific diets, dietary patterns, and supplements in preventing neurodegenerative disorders. It examines clinical studies that evaluate these interventions, highlighting the pharmacological basis of antioxidant-rich diets in managing and preventing these diseases. Additionally, this review explores functional foods like vitamins, fruits, vegetables, and specialized diets that may aid in managing neurodegenerative conditions. It concludes with insights into the clinical status of these functional foods in relation to such disorders.

## 2. Pharmacological Considerations in Brain Disorders

Mitochondrial function and energy production play a crucial role in regulating brain activity. Even in the resting state, the brain consumes around 20% of the body’s overall energy [66]. Thus, the performance of the brain is heavily governed by mitochondrial energy production, which supports brain activity, regulates neuronal excitability, modulates inflammation, and releases neurotransmitters [67]. An insufficient supply of ATPs can also lead to brain cell death. Further, mitochondrial function also influences behavior, mood, and cognition [68]. Mitochondrial function tends to decline with age, which is related to reduced efficiency of the electron transport chain, reduced ATP production, and increased ROS generation [66]. All these factors contribute to mitochondrial damage, leading to neuroinflammation. Changes in the food plans, such as limiting the consumption of processed foods and promoting the consumption of fruits, legumes, seeds, and other high-fiber foods, can prevent and slow down the damage. In this discussion, we explore potential pharmacological considerations in brain disorders, aiding in understanding the mechanisms and planning diets to modulate these functions.

### 2.1. Mitochondrial Dysfunction in Brain Disorders

**Figure 1:**
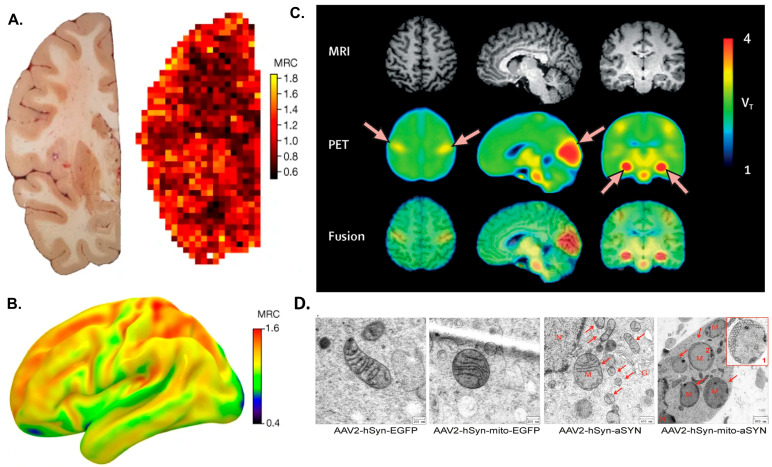
Mitochondrial brain mapping, mitochondrial dysfunction, and damage. (**A**) Distribution of mitochondria across the human brain in a 51-year-old man [76]. The voxels showing brighter color show the presence of mitochondria that are specialized for energy transformation. (**B**) Mitochondrial map and respiratory capacity across the brain. (**C**) PET imaging of neuroinflammation indicating COX1 expression in the human brain [78], which is produced in response to inflammation and expressed in most tissues. (**D**) TEM images showing distorted and fragmented mitochondrial cristae in response to wild-type α-synuclein overexpression [78]. Fragmented mitochondria are marked by red arrows. M. Neurons targeted with α-synuclein show swollen mitochondria with deformed cristae (G in the image indicates Golgi apparatus, N indicates the nucleus, and M indicates mitochondria).

Mitochondrial dynamics include fission, fusion, motility, and mitophagy, which are critical for mitochondrial homeostasis. Dysregulation in mitochondrial dynamics can be a key pathogenic mechanism in various diseases, including neurodegenerative diseases. Under normal conditions, an equilibrium exists between ROS production and their detoxification. This ensures that while ROS are generated as byproducts of cellular metabolism, they are efficiently neutralized, preventing any potential damage to cellular structures and maintaining overall cellular health. A shift in mitochondrial ROS production in turn causes oxidative stress, which causes irreversible damage to the mitochondrial membrane. Overproduction of ROS damages the cellular structure, contributes to oxidative stress, mitochondrial dysfunction, and impairs cellular functions [69]. Metabolic stress causes activation of multiple pathways in mitochondria, causing mutations in mitochondrial DNA [70]. These alterations disrupt normal cell functions, reduce biogenesis, and result in impaired mitophagy [71]. These mitochondrial abnormalities include age-dependent accumulation of mitochondrial DNA changes, increased mitochondrial ROS production, altered mitochondrial membrane potential, mitochondrial fragmentation, and enzymatic activities [72]. The excessive influx of calcium into mitochondria triggers mitochondrial fission and synaptic dysfunction [73]. Another factor is hypoxia, which reduces ATP production and damages brain cells [74]. Further, impaired oxidative phosphorylation and compromised ATP production are related to the development of neurodegenerative diseases [73]. Additionally, inflammation and inflammatory mediators like chemokines and cytokines induce mitochondrial dysfunction, contributing to neuronal damage [75]. Recently, a study reported a distribution of mitochondria (Mitochondria map) and respiratory capacity (Figure 1A,B) across the human brain of a 51-year-old man [76]. One study showed the expression of COX1 in the human brain using PET imaging (Figure 1C), which is an indicator of neuroinflammation and is produced in response to inflammation in most tissues [77]. Another study showed that α-synuclein (α-syn) promotes dopaminergic neuron dysfunction, a major hallmark of PD. The neurons with overexpressed wild-type α-synuclein showed distorted and fragmented mitochondrial cristae (Figure 1D). Further, mitochondria were swollen with deformed cristae in Transmission electron microscopy (TEM) images [78]. Alterations in mitochondria-associated endoplasmic reticulum membranes and mPTP (mitochondrial permeability transition pore) are interconnected with AD pathology, where Aβ-protein and hyperphosphorylated tau proteins interfere with mitochondrial function [79]. Overactivation of mPTP is linked to mitochondrial dysfunction and the development of AD [80]. In AD, defective autophagy-lysosome pathway impairs mitochondrial function and damages the CNS [81]. PD has also been linked to dysfunction of mitochondrial complex I. On the other hand, HD displays increased oxidative stress and dysfunction of mitochondria. Thus, it is essential to maintain mitochondrial health within brain cells (e.g., glial cells) for the proper functioning of neurons. Clearance of damaged mitochondria and maintaining cellular homeostasis are important for neurological health and overall well-being. Skawratananond et al. summarized multiple pharmacological agents (e.g., urolithin A, spermidine, resveratrol, and kinetin) classified as mitophagy modulators. These modulators have the potential to restore mitochondrial function [71]. Among specific nutrients, the role of vitamin B, tocopherol, ascorbic acid, coenzyme Q10, melatonin, and lipoic acid has also been reported in mitochondrial function [75,82]. The latter section discusses mitophagy modulators, diets, and supplements that can promote mitochondrial structural buildup.

### 2.2. Mitophagy

Mitophagy is a cellular protective mechanism to clear dysfunctional mitochondria, which involves lysosomal degradation of mitochondria for the recycling of cellular components. Damaged mitochondria are removed through autophagy, which is a “waste disposal system”. Generally, damaged mitochondria are selectively encapsulated by autophagosomes, which in turn form mitophagosomes. These mitophagosomes are fused with lysosomes, forming mitophagolysosomes. The damaged mitochondria are then digested into phagocytic vacuoles.

The key regulatory pathways of mitophagy include PINK1-Parkin, AMPK-ULK1, PGC-1α-NRF-1/2 transcription factor A [83,84]. These pathways also play a major role in mitochondrial biogenesis. Moreover, cytosolic Parkin is depleted in patients with AD, leading to abnormal accumulation of PINK1. Further, the overexpression of Parkin tends to restore mitophagy.

Certain food ingredients act as mitophagy inducers and impact mitochondrial function in many ways. Such functional foods include polyphenols, such as urolithin, mangiferin, and morin, which protect the mitochondrial membrane potential, preventing the activation of caspases in neurons, thereby inhibiting apoptosis [71]. Similarly, specific diets such as a high-fat diet impair mitochondrial biogenesis and contribute to mitochondrial dysfunction [85,86]. The list of mitophagy inducers impacting mitochondrial and brain function is summarized in Table 1. In contrast, the ketogenic diet promotes mitochondrial efficiency, enhances oxidative phosphorylation, and increases ATP production [87]. Caloric restriction and intermittent fasting stimulate mitochondrial biogenesis, enhance autophagy, and promote cellular repair. The Mediterranean diet combines foods containing healthy fats and antioxidants that support mitochondrial health and improve its efficiency [88].

### 2.3. Glymphatic Clearance for Brain Health

Brain waste clearance is crucial for maintaining good neurological health. One such pathway involved in brain clearance is the glymphatic system. This system facilitates the drainage of cerebrospinal fluid and interstitial fluid towards the cervical lymph nodes. Aquaporin-4 (AQP4), a transmembrane water channel protein, is associated with the flow of fluids and the glymphatic system [102]. The reduction in the AQP4 perivascular localization has been shown to reduce the efficiency of the glymphatic clearance and contribute to the development of neurological disorders. The glymphatic system is also associated with the clearance of β-amyloid and α-synuclein, which are associated with the development of AD and PD [103,104]. Further, dysfunctional glymphatic clearance has been linked to neuroinflammation [104]. Delle et al. studied the long-term effect (10 months) of a high-fat diet on glymphatic flow in mice [105]. The 10-month exposure to a high-fat diet did not change the overall glymphatic flow; however, it enhanced the regional glymphatic flow in the hypothalamus. Results suggest that neuroprotective adaptations can occur with long-term exposure to a high-fat diet, causing no observed change in overall glymphatic flow. Additionally, AQP4 perivascular localization was increased in the hypothalamus region [105]. Further, obesity has been shown to significantly impact brain energy use and metabolic expenditure, leading to alterations in glymphatic flow within the hypothalamus. The glymphatic system is known to play a major role in the clearance of such proteins from the brain to reduce the risk of the development of AD [106].

Recent studies have shown the impact of diet and supplements (e.g., omega-3 fatty acids) in supporting brain clearance via the glymphatic system. Supplementation with PUFAs has been shown to increase the clearance of β-amyloid protein via the AQP4-dependent glymphatic system [107]. Specific supplements (e.g., β-hydroxybutyrate) exhibit neuroprotective effects by promoting glymphatic function [108]. A study by Yao et al. used a chronic unpredictable mild stress mouse model of depression [109]. These mice had depressive behaviors and impaired glymphatic function due to expression of AQP4 in perivascular astrocytes [109]. Melatonin supplementation in this mouse model improved glymphatic function. Thus, it is evident that the role of diet and specific supplements in supporting brain health is crucial.

### 2.4. Neuroinflammation

Neuroinflammation is a multifaceted pathophysiological response of the body and brain in response to any injury or disease. As discussed in an earlier section, mitochondrial dysfunction and neuroinflammation are linked, which is greatly influenced by diet and habit. For example, a high-fat diet induces neuroinflammation. It can increase the permeability of the BBB, which in turn can allow proinflammatory factors to interact with glial cells, promoting inflammation and neurodegeneration [110,111]. Studies have reported that if proinflammatory cytokines and chemokines cross the BBB, they can trigger microglia and immune cells in the CNS [112]. This can result in neuroinflammation, which in turn contributes to synaptic dysfunction and cognitive decline. Systemic inflammation and neuroinflammation share common inflammatory pathways, including NF-kB, IL-6, and TNF-α. A study discussed in an earlier section showed the expression of COX1 in the human brain using PET imaging (Figure 1C). COX1 is an enzyme and a common indicator of inflammation, which is produced in response to inflammation in most tissues [77]. Healthy food habits are often linked to lower levels of neuroinflammation and a reduced risk of neurodegeneration. Diet, meal timing and frequency, caloric restriction, and fasting also influence brain aging [113]. Impact of caloric restriction on brain health is discussed in a later section.

### 2.5. Brain–Body Interactions in Brain Health

#### 2.5.1. Gut–Brain Axis, Microbiome, and Virome

**Figure 2:**
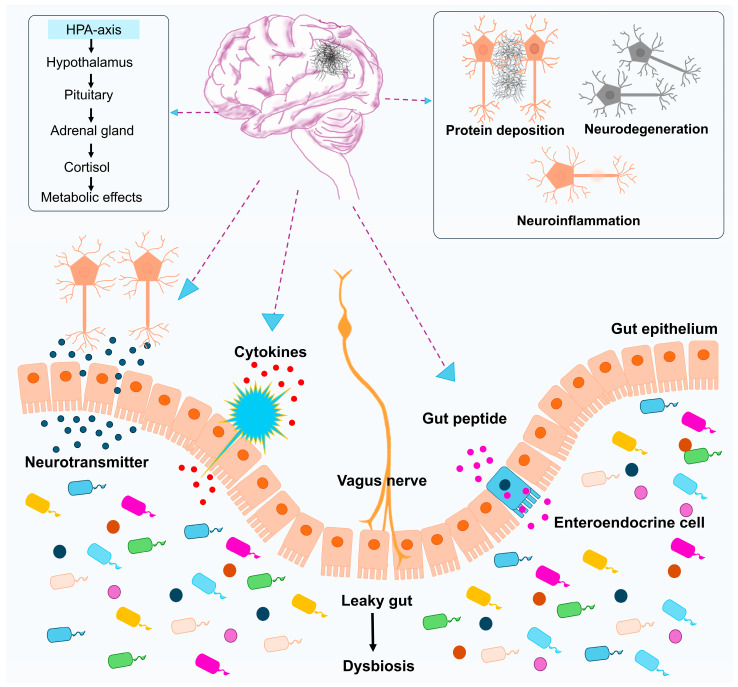
Schematic of the gut–brain axis and key mechanisms that play roles in brain health and neurodegeneration.

The gut–brain axis is a bidirectional communication that connects the gastrointestinal tract to the CNS [114]. The gut–brain axis includes the CNS, autonomic nervous system, Enteric nervous system (ENS), immune system, and hypothalamic–pituitary–adrenal axis (Figure 2) [115,116]. This intricate system is associated with neural, hormonal, and immunological signaling [117,118]. A well-balanced healthy gut microbiota maintains intestinal barrier integrity and influences the brain through neurotransmitter production, vagus nerve stimulation, and production of neuroactive metabolites [119].

The gut–brain axis influences metabolic, neural, endocrine, and immunological pathways (Figure 2). Among neuronal pathways, the vagus nerve extends from the brainstem, intervening in the gut and ENS. Gut microbiota interacts with the hypothalamic–pituitary–adrenal axis and regulates brain function along with gut microbiota composition. Metabolites, including neurotransmitters, amyloids, and SCFAs, reach the brain and regulate neuronal networks. Dysbiosis in gut microbiota composition leads to dysfunction in the intestinal barrier and correlates with the development of neurodegenerative conditions.

The gut microbiota comprises around 100 trillion microorganisms that engage in a symbiotic relationship. About 90% of the total community of microbes in the human gut is the Firmicutes and Bacteroidetes Phyla [120]. These microorganisms have unique abilities [121], including the synthesis of neurotransmitters. For example, *Escherichia coli* and Enterococcus produce serotonin. Gut microbes process these foods, generating metabolites from amino acids, polyphenols, and polysaccharides, which play crucial roles in brain functions [122]. Gut bacteria tend to encode various gene pathways that tend to metabolize aromatic amino acids (e.g., tyrosine, tryptophan, and phenylalanine) into tyramine, dopamine, adrenaline, and noradrenaline. These molecules can cross the BBB and contribute to brain functions. Further, dietary fiber is not digested in the human gut but is instead fermented by gut microorganisms, producing Short-chain fatty acids (SCFAs), including butyrate, acetate, and propionate. These SCFAs have been shown to improve symptoms of multiple sclerosis [123]. Alterations in gut microbiota composition is commonly known as dysbiosis [124]. It is implicated in developing autoimmune, metabolic, gastrointestinal, and neurodegenerative diseases [125].

The gut virome relation to brain health has received attention in recent studies, which includes bacteriophages [126,127,128]. Implications of variable virome composition have been linked to neuropathological hallmarks of Parkinson’s disease [129,130]. Recent studies suggest that α-syn aggregation may begin with bacterial nitrate reduction in the gut [131]. Stockdale et al. studied the effect of α-syn alterations on gut virome diversity over 5 months [132], which showed significant changes in virome biodiversity. However, further studies are required for clear establishment.

Traditionally, the appendix has been viewed as a vestigial organ with no significant purpose [133,134,135,136]. However, recent studies suggest that the appendix plays a role in the immune system, which is linked to brain function [121]. It contains lymphoid tissue that contributes to immune responses to maintain brain health by preventing inflammation and infections that might adversely affect neurological function. It acts as a reservoir for the microbiome and virome, helping reboot the microbiome after disruptions. Ongoing research is examining the links between appendix health, gut health, and neurodegenerative diseases like Alzheimer’s and Parkinson’s [133,134]. Understanding these connections could lead to new insights into preventative measures for brain health. Further research is needed to fully understand the relationship between the appendix, gut health, and brain health.

Dysfunction of the intestinal barrier enhances the risk of systemic exposure to endotoxins, leading to the development of such chronic conditions [137]. Ultra-processed foods (e.g., western diets) are abundant in saturated fats and refined carbohydrates, which negatively affect gut microbiota composition and brain health [138]. Intake of the ketogenic diet produces ketone bodies that promote beneficial changes in the brain and gut microbiota [139]. This includes a decrease in the level of Proteobacteria and actinobacteria, and an increase in SCFAs. Whereas in the brain, an increase in the ketone body increases the synthesis of MCT receptors on the BBB, which enhances brain uptake of ketone bodies [140]. Ketone bodies act as an alternative cerebral fuel, restoring the brain’s energy demand by enhancing ATP production. They also decrease neuroinflammation by decreasing chemokines, cytokines (IL-1β, IL-6, and TNF-α), microglia activation, and NLRP3 (NOD-, LRR-, and pyrin domain-containing protein 3) Inflammasome [141]. Further, the interaction between gut microbiota and immune system plays a critical role in multiple sclerosis. In the gut, plasma cells are known to produce large amounts of IgA antibodies. Recently, Probstel et al. mentioned the role of gut-derived IgA+ B cells, which attenuate inflammation in the CNS, mainly in multiple sclerosis [142]. Treatment with faecal microbiota transplantation re-establishes the gut microbiota and reduces proinflammatory microbial products like lipopolysaccharide (LPS). This reduces systemic inflammation by decreasing intestinal barrier permeability to potential toxins and limiting the exposure of microbes, proinflammatory cytokines (IL-1β, TNF-α, and IL-6), and LPS to the systemic circulation. It also reduces their access to the brain [143,144].

Also, treatment with faecal microbiota transplantation in patients with PD has been shown to reduce constipation (non-motor symptoms) by providing bacterial strains such as Faecalibacterium prausnitzii, which produces SCFAs. These act as anti-inflammatory agents, increase ENS activity, and promote gastrointestinal motility [144]. A study in pregnant mice suggested the production of inflammatory cytokines like IL-17 due to the presence of filamentous bacteria in the gut. Further, these bacteria can cross the placenta and enter the foetus’s brain, causing autism-like behavior [145]. Another study suggested that the bacterium Akkermansia muciniphila produces vitamin B3 in the gut, which travels to the brain and reduces motor neuron disease symptoms (e.g., ALS) [146].

#### 2.5.2. Heart–Brain Axis

The heart–brain axis is also a bidirectional communication that involves neural, humoral, and immunological pathways. Further, several studies have emerged showing cardiac dysfunction correlation with neurodegenerative diseases [147,148]. Oxidative stress and hypoxia are major contributing factors [149], affecting cerebral arterioles, which contribute to the development of small vessel diseases such as arteriosclerosis and cerebral amyloid angiopathy [150]. Brain-derived neurotropic factor (BDNF) has emerged as an important biomarker for cardiovascular diseases, explaining the heart–brain axis [151]. BDNF also plays a role in angiogenesis and supports brain health.

Exercise stimuli can prevent or slow cognitive decline in elderly patients with heart failure. In this respect, vagal stimulation, exercise training, electrical neurostimulation, and music therapy have become interesting options in the treatment of angina pectoris, heart failure, and hypertension, which in turn improves neurological conditions [148].

#### 2.5.3. Other Organs

In addition to the gut–brain and heart–brain axis, other organs also exhibit bidirectional communication with the brain. For example, CNS inflammation is linked to liver function [152], where liver disease and dysfunction can affect cognition. A clinical study performed MRI scans, cognitive testing, and plasma biomarker analysis in patients with liver disease. The liver disease accelerated total brain volume and regional brain volume loss, mainly in the frontal and temporal white matter [153]. The patient, who has a documented history of hepatitis, exhibited a notable decline in verbal fluency, with an increase in neurofilament light levels. This raises concerns about the potential impact of liver condition on cognitive function. 

Liver X receptors are nuclear receptors that are key regulators of lipid metabolism and inflammatory responses [154]. Activation of liver X receptors can regulate neuroinflammation and reduce the accumulation of β-amyloid. The liver–brain axis can also result in neuroinflammation via activation of microglia. Western diets are known to induce metabolic and systemic disturbances in the liver, along with neuropathological changes in the brain, leading to the development of AD [58]. High consumption of Western diets results in hypercholesterolemia, which is also related to the onset of AD.

### 2.6. Vagus Nerve Stimulation Impacted by Food

Vagus nerve stimulation (VNS) was approved in 1994 as an adjuvant therapy for refractory epilepsy. Multiple studies have reported reductions in seizure frequency and improvements in cognition after VNS stimulation [155]. The vagus nerve has a significant role in the reflex control of physiological homeostasis and in communication between the gut and brain. Alteration in gut microbiome can impact the functioning of the vagus nerve, which can lead to impaired cognition, depression, and anxiety [119]. Diet and certain foods that support vagus nerve function and stimulation can influence neurodegeneration through gut–brain signaling mechanisms. VNS also influences the production of BDNF and influences inflammation. Various studies have also implicated that VNS can reduce serum cortisol levels and prevent Alzheimer’s disease by restricting stress severity [156]. Increasing evidence indicates that food can influence cognition through the VNS [157]. For example, foods rich in omega-3 fatty acids, tryptophan, prebiotics, and probiotics have been shown to influence vagus nerve activity [158]. Other reports also suggest a positive correlation between the Mediterranean diet and vagal tone [158].

### 2.7. Ferroptosis

Ferroptosis cell death is characterized by iron-dependent lipid oxidation, accompanied by variations in cell morphology and protein expression [159]. Growing evidence suggests a correlation between ferroptosis and brain function [160]. The brain contains high levels of PUFA, which are the major lipid peroxide precursors. Multiple studies have reported dysregulation of iron homeostasis, ROS accumulation, and inactivation of antioxidant systems, all of which are responsible for the progression of neurodegenerative diseases [161]. This underscores the role of ferroptosis in the pathogenesis of AD and PD. Specific dietary components can influence ferroptosis through modulation of iron metabolism and lipid peroxidation. Such dietary components, including resveratrol, curcumin, quercetin, and α-lipoic acid, can inhibit ferroptosis [162,163]. Specific diets, including the ketogenic diet, can also alleviate brain iron deposition, impacting cognitive function [164]. The impact of the ketogenic diet on brain health is discussed in detail in a later section.

### 2.8. Impact of Caloric Restriction on Brain Health

Caloric restriction (CR) is a lifestyle intervention that improves long-term brain health owing to enhanced cognitive function. Physiological alterations associated with CR and fasting are known to have profound implications linked to dementia and reduced β-amyloid accumulation [165]. CR prevents the synthesis and deposition of β-amyloid protein by facilitating its proteolysis. Further, intermittent fasting can increase ketone body metabolism and β-hydroxybutyrate levels in the blood. Further, β-hydroxybutyrate has been shown to exert protective effects against AD [165,166]. β-Hydroxybutyrate has also shown antioxidant properties, protection against oxidative damage, reduced inflammation, and promotion of cellular health. Other studies on fasting and ketogenic diets suggest that CR reduces seizures and imparts anticonvulsant effects [167]. In patients with multiple sclerosis, a 22% reduction in calorie intake correlated with lower depressive scores, which in turn improves quality of life in patients [168]. Further, CR causes upregulation of sirtuin proteins, which reduce neuronal loss, maintain cellular metabolism, stimulate antioxidant activity, and inhibit inflammatory pathways [167]. Despite the reported benefits of CR in improving quality of life, adherence to routines is challenging for many people due to lifestyle. Therefore, calorie restriction mimetics (CRMs) were developed. CRMs are natural polyphenolic compounds with similar biochemical and molecular effects mimicking CR. Other than these, glycolytic inhibitors like d-glucosamine, polyamines, and NAD+ precursors also function as CRMs. Such CRMs regulate redox signaling through activation of the Nrf2 pathway and attenuation of mitochondrial function, as well as PI3K/Akt and MAPK pathways [169]. CRMs like resveratrol, chlorogenic acid, and rosmarinic acid, derived from caffeic acid, improve age-related disorders [169]. Glucosamine has also been reported to activate AMPK and inhibit glycolysis, inducing mitochondrial biogenesis [170]. CRMs also activate autophagy and prolong life span. Further, CRMs can block the electron transport chain and activate AMPK. This helps delay signs of aging and reduces instances of age-related chronic diseases.

## 3. Diets and Their Mechanism in Preventing Neurodegenerative Diseases

People with neurodegenerative conditions are at greater risk of developing malnutrition [171]. Specific dietary patterns and nutrients, such as antioxidants, vitamins, omega-3 fatty acids, and fiber intake, are crucial for preventing cognitive impairments [172]. These dietary components promote neurological health by preventing oxidative stress, neuroinflammation, and many other pharmacological effects, as discussed in the earlier section [173,174,175]. Recent studies highlight a complex relationship between inflammatory pathways in the brain and nutrient metabolism [176]. Specific diets, including the Mediterranean diet, Modified Atkins diet, Paleolithic diet, and Swank diet, have been shown to prevent incidences of neurodegenerative diseases [177] and improve the quality of life of patients.

### 3.1. Foods That Worsen Neurodegenerative Diseases

Unhealthy, unbalanced diet, along with environmental factors, including smoking, stress, alcohol consumption, pesticide exposure, and a sedentary lifestyle, are significant causes of the progression of neurodegenerative diseases [178]. An unbalanced diet during the pre-conception phase, pregnancy, and the first few years of childhood causes epigenetic alterations that promote neurodegeneration [179]. Unhealthy dietary habits of mothers can also alter the gut microbiota of neonates via breastfeeding, which risks the development of neurodegenerative diseases [180,181].

Consumption of whole grains can affect cognitive domains during childhood and adolescence [182]. In adults, the consumption of rye bread has been shown to improve glucose tolerance and postprandial insulin response, which also improves memory [183]. However, in children around 10 years of age, whole-grain consumption was not associated with any cognitive domains [184]. Similarly, the consumption of dairy products has been shown to improve cognitive functions in young children. But milk intake in the case of older adults was negatively correlated with verbal memory performance [185]. Casein is the main milk protein, of which 30–35% is β-casein [186]. Two variants of β-casein are A1 and A2, which differ by only one amino acid. Evidence suggests that consumption of A1 β-casein is associated with developing coronary artery disease, type-1 diabetes, schizophrenia, and autism [187]. Some studies also suggest that A1 β-casein increases gastrointestinal inflammation and worsens post-dairy digestive discomfort [188].

Consumption of refined sugars and sweeteners, including sweetened beverages, adversely affects cognitive functions, especially during childhood and pregnancy [189]. While low consumption of saturated fats improved cognitive flexibility, high consumption can cause impaired memory and control [190]. Further, these simple sugars increase intracellular prooxidants and can cause alterations in the gut microbiota, which are correlated with the development of neurological conditions [191]. Other reports have suggested that consumption of a high-fat diet or high-fat and high-sucrose (HFHS) diet, reduces the level of vitamin C in the frontal cortex, which is essential for normal brain function [192]. A randomized study design evaluated the relation between milk intake and AD, PD, MS, and ALS. They have indicated that genetically predicted milk consumption increased the risk of PD [193]. However, this study did not specify the type, content, or sources of milk that can cause these issues. Sweeteners, dietary products, and red meat are also associated with cognitive impairment [194]. Ylilauri et al. included 2497 participants listed as dementia-free men aged 42–60 from Eastern Finland [195]. They investigated the association between dairy, fish, meat, and cognitive performance, which showed that 337 men reportedly had dementia, worsened verbal fluency, and visual impairments [195]. Consumption of Annona muricata fruit (Soursop) has become a risk factor that can worsen the severity of these diseases [196]. Earlier, fruits, juices, or herbal teas from the Annonaceae family were widely consumed in the Caribbean for their traditional medicinal properties and taste. However, experimental evidence indicates that it could lead to PD and other neurological manifestations.

### 3.2. Diets for Prevention and Management

Now, we understand that dietary choices significantly impact physiology and the prevention and management of diseases. Various dietary patterns have been developed and studied, showing a significant role in maintaining overall health. Among these, the Paleolithic diet, Mediterranean, MIND, and ketogenic diets have gained attention for managing such health conditions. Some of these emphasize whole foods reminiscent of our ancestral eating habits, some focus on rich flavors and heart-healthy ingredients, and some uniquely combine elements of other diets to support brain health. In contrast, the ketogenic diet is recognized for its low-carb, high-fat approach. Mediterranean, ketogenic, and vegan diets have been shown to improve cognitive symptoms and reduce β-amyloid protein [197]. Overall, these dietary frameworks contribute to overall health and the management of disease conditions.

#### 3.2.1. Paleolithic Diet

The Paleolithic diet is a modern concept of the diet consumed by humans during the “Old Stone Age” or Paleolithic era [198,199]. It includes the consumption of a diet based on plants, animals, seafoods, and insects but excludes grains, fibers, and legumes [200,201]. A Paleolithic diet is beneficial in cases of type 2 diabetes and cardiovascular effects [202]. Such dietary restrictions have enhanced functional brain responses in the anterior hippocampus, strongly linked to increased BDNF that mediates neurogenesis and synaptic formation [203,204]. Paleolithic diet has been shown to reduce the risk of developing metabolic syndromes [205]. A modified Paleolithic diet, also known as the Wahls elimination diet, excludes grains and dairy while encouraging the consumption of fruits and vegetables, which has been used for specific neurological conditions [206]. Various clinical studies (NCT01381354 and NCT03659422) are exploring the benefits of the Paleo diet in neurodegenerative conditions.

#### 3.2.2. Mediterranean Diet

The Mediterranean diet includes the consumption of vegetables, fruits, cereals, seeds, and nuts as major sources of fats. It also includes moderate consumption of dairy products and low processed meats. Adherence to the Mediterranean diet has been shown to improve cognitive functions [207,208]. Several clinical studies have indicated benefits of the Mediterranean diet in neurodegenerative conditions, including MS (NCT05175378), PD (NCT03851861), and AD (NCT04435509 and NCT04440020). Specific compounds in the Mediterranean diet act as caloric restriction mimetics and impart neuroprotective effects [209]. It has been shown to reduce the risk of stroke, neurodegenerative diseases, and vascular dysfunction [210,211]. Administration of extra virgin olive oil in mouse models with β-amyloid deposition reduced β-amyloid and tau proteins, improving cognitive performance [212].

#### 3.2.3. DASH Diets

The Dietary Approaches to Stop Hypertension (DASH) [213] is a pattern that identifies dietary factors that affect blood pressure and prevent hypertension [214]. These diets are rich in fruits, vegetables, nuts, low-fat dairy products, lean meat, and whole cereal products. Adherence to the DASH and Mediterranean diets has been associated with slow cognitive decline [215]. Although the Mediterranean/DASH/MIND diets are plant-based, the significant difference lies in the type of food and the recommended levels [216]. The DASH diet aims to provide benefits in controlling blood pressure, preventing cognitive decline, and preventing diabetes [217]. The other benefits of DASH diets are reducing the risk of colorectal cancer, chronic liver disease, and managing chronic heart failure [218,219].

#### 3.2.4. MIND Diet

MIND stands for a hybrid of the DASH and Mediterranean diets, an intervention for the neurodegenerative delay [220]. It includes the consumption of plant-based foods, mainly berries and leafy vegetables, specifically designed to support the brain. The MIND diet limits the intake of animal-based products and foods with a high saturated fat content, which can enhance β-amyloid protein levels [221,222]. The MIND diet reduces the risk of developing cognitive dementia. This diet works by reducing inflammation, oxidative stress, and β-amyloid deposition [223]. Various clinical studies (NCT04337255, NCT02817074, and NCT05301868) have explored the benefits of the MIND diet in neurodegenerative conditions.

#### 3.2.5. Ketogenic Diet

It is a fat-rich, low-carbohydrate diet with fasting-like effects, which lead to a state of ketosis. It is known to reduce appetite and shows gastrointestinal side effects, which is referred to as keto flu. It has shown benefits in epileptic seizures, ALS, cerebral ischemia, AD, and PD [224,225]. Ketogenic diets can increase ketone body production levels and reduce blood glucose concentration [226]. Further, these ketone bodies pass through the BBB and enter the brain to replace glucose as an energy fuel for neurons, which in turn inhibits the hyperexcitability of neurons to prevent seizures [227]. The ketogenic diet has also been shown to improve motor and non-motor symptoms in people with PD [228]. Various clinical studies (NCT05152771, NCT00777010, and NCT05152771) have shown the benefits of a ketogenic diet in neurodegenerative conditions.

#### 3.2.6. Modified Atkins Diet

The modified Atkins diet, often called “MAD”, is quite like the traditional ketogenic diet but is restrictive. It was initially developed in the 1970s for weight loss, but it is now “modified” from Atkins diet. Also, there is no caloric restriction; instead, fats and proteins are encouraged [229]. The modified Atkins diet has been helpful in refractory epilepsy [230]. A study evaluated MAD’s safety, efficacy, and tolerability in 14 refractory epilepsy cases among Korean children [231]. After 6 months, only 50% (7 subjects) adhered to the diet, 36% showed over 50% reduction in seizures, while 21% were seizure-free. Various clinical studies (NCT03718247, NCT03585907, and NCT02521818) have shown the benefits of the modified Atkins diet in neurodegenerative conditions.

#### 3.2.7. Swank Diet

The Swank diet is based on evidence that high-fat consumption, mainly from dairy and meat sources, has a higher incidence of MS [232]. It is a diet with low-fat, specifically low saturated fat, that can reduce the severity of symptoms and frequency of flares in case of MS [233]. A recent study demonstrated that a low-saturated-fat diet or the Swank diet can improve recognition, reduce fatigue in relapsing MS, and improve the quality of life [232]. Dr. Roy Swank introduced the Swank Diet in 1948. Swank placed patients on his recommended diet, including consuming nearly 20g of saturated fats per day. Results indicated lower mortality and less disability [234,235]. Various clinical studies (NCT02914964) have reported the benefits of the Swank diet in neurodegenerative conditions, including MS.

## 4. Clinical Studies on the Management of Neurodegenerative Disease with Diet Intervention

### 4.1. Parkinson’s Disease

PD involves continuous degeneration of neurons in the basal ganglia, causing motor dysfunctions [236]. Symptoms include uncontrolled movements such as shaking of the limbs or jaws, body stiffness, loss of cognition, sleep disturbances, slurred speech, and behavioral changes [237]. It also involves non-movement symptoms like fatigue and irregular blood pressure [238]. Usually, PD affects males in their early sixties more than females [237].

The impact of diet in slowing down the progression of PD has emerged as a potential strategy for addressing such neurodegenerative diseases [239,240]. Specific diets and supplements have shown improvements in cognitive outcomes. Some of the mechanisms and outcomes of the specific diets for PD are summarized in Table 2. The Mediterranean diet includes a high intake of fruits, legumes, vegetables, unsaturated fatty acids, and fish. The diet contains bioactive compounds like vitamin E, vitamin C, β-carotene, omega-3 fatty acid, protein, and polyphenols, which act as antioxidants and anti-inflammatory agents by downregulating inflammatory mediators like C-reactive protein, IL-6, and fibrinogen [241]. Dietary intake of vitamin E and β-carotene lowers the risk of PD [242]. Punicalagin is an ellagitannin from pomegranate, which is useful in PD [243]. It is known to prevent dopamine oxidation, reduce oxidative stress, and downregulate inflammation, exerting neuroprotective effects [244]. Besides PD, it is also beneficial in MS [245].

**Figure 3:**
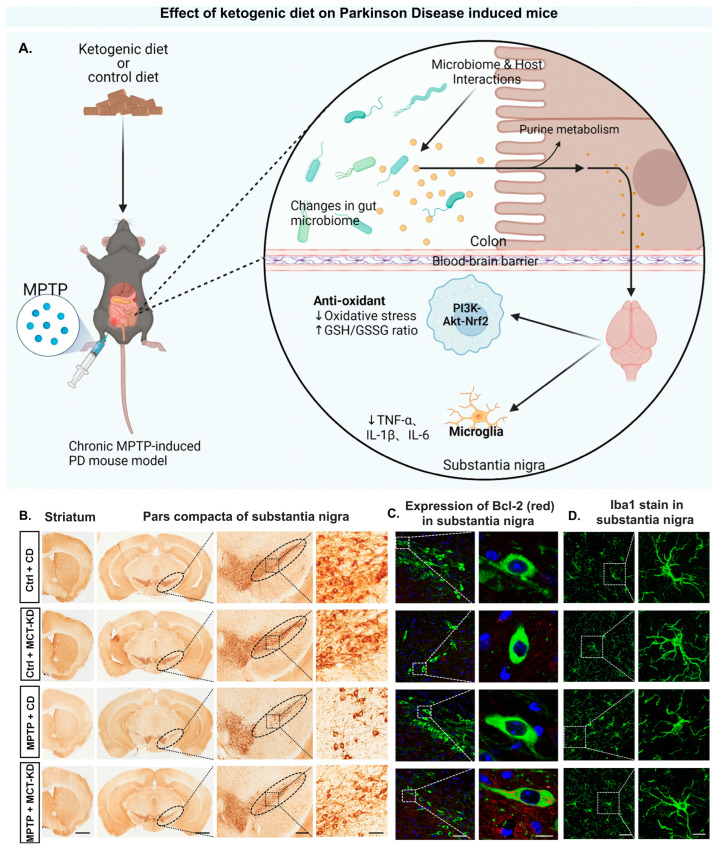
(**A**) Schematic showing PD induction in mice using 1-methyl-4-phenyl-1,2,3,6-tetrahydropyridine (MPTP) and studying the effect of ketogenic diet in PD mice. (**B**) Histology images of the mouse brain after immunohistochemical staining for tyrosine hydroxylase-positive neurons in brain regions after different diets. Darker color indicates higher levels of tyrosine hydroxylase. The ketogenic diet reduced tyrosine hydroxylase levels. (**C**) Immunofluorescent images of neurons with the anti-apoptotic protein (Bcl-2, red color) in the substantia nigra region of the brain, which is increased in mice fed the ketogenic diet. (**D**) Neuroinflammation study using microglial specific marker to observe morphology, where the ketogenic diet reversed the morphological changes induced in PD mice. Reprinted (adapted) under the terms of the Creative Commons CC license from [248].

Intake of the Mediterranean diet elevates SCFAs such as acetate, butyrate, and propionate levels, stimulating gut microbiota like Roseburia spp. This helps maintain the intestinal barrier and reduce inflammation, leading to decreased non-motor symptoms (constipation) associated with PD [246]. Studies show that Mediterranean diet consumption protects against alpha-synuclein aggregation and exerts anti-inflammatory action and antioxidant activities [247]. Furthermore, improvements in the gut-to-brain signaling pathway reduce early symptoms of prodromal PD like constipation, depression, and daytime drowsiness. Figure 3 presents studies involving the impact of the ketogenic diet on PD-induced mice. Figure 3B shows neuroprotective effects on dopaminergic neurons upon long-term use of MCT-KD (medium chain triglyceride-ketogenic diet). Compared to the MPTP + CD group, the MPTP + MCT-KD group showed upregulation of the anti-apoptotic protein BCl-2 and prevention of dopaminergic neuron loss (Figure 3C).

A meta-analysis states that probiotics rehabilitate gut microbiota, promote bowel movement, and provide beneficial strains like *Lactobacilli* and *Bifidobacteriu*, preventing disease-associated constipation [249]. Probiotic supplementation containing bacterial strains like *L. reuteri*, *L. fermentum*, *L. acidophilus*, and *B. bifidum* reduces free radicals, oxidative stress, and inflammatory mediators (interferon-gamma and IL-6). It potentiates clinical and biochemical profiles in people with PD [250]. An ovo-lacto vegetarian diet containing ghee, vegetables, fruits, milk, and eggs provides bioactive compounds such as SCFAs, which provide a neuroprotective and anti-inflammatory effects. It improves gut microbiota and promotes bowel cleaning, which reduces constipation and motor symptoms associated with the disease [251]. Intake of a diet containing whey protein provides bioactive Leucine and vitamin D to the body. It boosts muscle protein synthesis, improving muscle mass and motor function accompanying PD [252].

Adherence to specific healthy diets, including Mediterranean, Modified Atkins, ketogenic, and gluten-free diets, is associated with specific components that have beneficial effects on the body. Specific diet supplements include vitamin D3, vitamin B6 and B12, coenzyme Q10, MitoQ, VIUSID/ALZER, Fortiral, nano-PSO, PS128, Betaquik MCT supplement, and Mind Master impact the development and progression of PD. Customized diets and supplements like mind master (NCT02837107) include Aloe barbadensis miller gel, Polygonum cuspidatum extract, grape juice, green tea extract, vitamin B1, vitamin B12, vitamin E, ascorbic acid, and folic acid. Supplements like Ethnodyne visiois are a plant-based food supplement with vitamin B2, and Supressi is a high-protein oral supplement for managing neurodegenerative conditions (NCT01192529). Creatine is another widely used supplement demonstrating neuroprotective effects in animal models of PD [253]. Similar trials with the effect of creatine supplementation on patients with PD are ongoing (NCT00449865). These food supplements, combined with drug therapies, improve overall patient health. Table S2 lists clinical studies associated with dietary foods and supplements in PD. This also represents the data based on different age groups and clinical phases (1–4), and Early Phase 1 is listed as Phase 0.

**Table 2:** List of studies showing diets for PD management with potential active compounds and their effects.

Food as Medicine	Mechanism of Action	Bioactive Compounds/Chemical Constituents	Outcome/Results/Efficacy	Ref.
Mediterranean diet containing vegetables (6 servings daily), fruits (3 servings daily), legumes (3–4 servings weekly), unsaturated fatty acids (olive oil), and fish (5–6 servings weekly).	Antioxidants and anti-inflammatory effects.	Vitamin C, vitamin E, β-carotenoids, protein, polyphenols, and omega-3-fatty acids.	Improves total antioxidant serum levels and prevent neurodegeneration.	[241]
Dietary antioxidants.	Counteract oxygen free radicals, thus decreasing oxidative damage.	Vitamin E and β-carotenoids.	A diet containing antioxidants is associated with fewer incidences of PD.	[242]
Nutritional Diet containing whey protein twice daily for 30 days.	Potentiate Muscle protein synthesis.	Leucine and vitamin D.	Increases muscle mass and improves motor function.	[252]
Probiotics.	Rehabilitate gut microbiota, and promote bowel.	*Lactobacilli* and *Bifidobacterium*.	Improves constipation and alleviates symptoms of PD.	[249]
Probiotics (2 × 10^9^ CFU/g for 12 weeks).	Antioxidants and anti-inflammatory; modulate insulin resistance.	*L. reuteri*, *L. fermentum*, *L. acidophilus*, and *B. bifidum*.	Potentiate clinical and biochemical profiles in people with PD.	[250]
Mediterranean diet.	Antioxidant, anti-inflammatory, neuroprotective, and improve gut–brain signaling pathway	Protein, carbohydrate, lipid, and alcohol.	Consumption of the Mediterranean diet reduces early symptoms of prodromal PD.	[247]
Mediterranean diet.	Improve bowel movement and intestinal barrier function and decrease inflammation.	Short-chain fatty acids (butyrate, propionate, and acetate).	Improves gut microbiota, constipation, and symptoms of PD.	[246]
Ovo-lacto vegetarian diet containing ghee, vegetables, fruits, nuts, seeds, milk, and egg products (3 meals per day).	Neuroprotective and anti-inflammatory.	Short-chain fatty acids (butyric acid and propionate).	Improves gut microbiota and bowel clearance, reducing motor symptoms associated with PD.	[251]

### 4.2. Alzheimer’s Disease

Alzheimer’s disease (AD) is a progressive neurodegenerative disorder characterized by dementia and other cognitive disabilities [26]. It causes the accumulation of β-amyloid proteins in the medial temporal lobe and neocortical regions [254]. It is a cognitive disorder with impaired memory, difficulties in reasoning or problem-solving, decreased thinking ability, and reduced daily functioning quality [255]. It has a higher prevalence in the elderly in their mid-sixties onwards. In 2020, approximately 5.8 million people were affected by AD, which is expected to grow to 16.4 million by 2060 [256]. AD is the most common type of dementia, and it develops by forming neuritic plaques and neurofibrillary tangles [257]. So far, no cure or effective prophylaxis for dementia has been reported. Consequently, dietary patterns are gaining attention in AD.

Intake of carotenoid-rich vegetables like carrots, sweet potatoes, red, green, and yellow pepper enhances circulating carotenoid levels, which has direct antioxidant benefits. Such diets in AD are summarised in Table 3. Specific diets, including the Mediterranean diet, rich in antioxidants, have shown to improve cognitive function [258]. Many other nutrients, including polyphenols, curcumin, vitamins B6 and B12, unsaturated fatty acids, and probiotic bacteria, can slow the course of AD [259]. The MAD is a low-carbohydrate, high-fat diet that induces ketogenesis and has been shown to improve episodic memory and cognitive abilities in patients with early AD [260]. A randomized study on the consumption of high-fat to low-carbohydrate MAD improved mild cognition and AD [260]. A randomized crossover trial stated that a ketogenic diet containing vegetables, oils, and nuts generates physiological ketosis, promoting improvements in brain cellular energy levels in AD [261] and improving cognition and quality of life in hospitalized patients with AD [261]. Consumption of a Mediterranean diet enriched with walnut extract has been shown to induce ATP production in mitochondria. It also reduces β-amyloid (1–40) generation and promotes neuronal functions [262]. These effects are attributed to walnut active constituents like oleic acid, linoleic acid, α-linolenic acid, and gamma- and beta-tocopherol. Clinical studies examining specific diets and other dietary supplements in AD are summarized in Table S3. Besides the Mediterranean and MAD, the MIND diet has been shown to improve cognition, enhance brain resilience, and provide benefits for AD-associated dementia in chronic patients [217,263,264]. It includes bioactive compounds (folate, vitamin E, lutein-zeaxanthin, and flavonoids), with potential anti-inflammatory and antioxidant properties.

Alterations in gut microbiome composition due to changes in dietary habits affect AD’s development and progression [265]. Intake of a probiotic supplement boosts gut microbiota with bacterial strains like *L. acidophilus*, *B. bifidum*, and *B. longum*, which exert antioxidant and anti-inflammatory (decreases serum high-sensitivity C-reactive protein) effects. This positively affects AD-associated cognitive ailments [250]. A meta-analysis on dietary intake of vitamin E, which constitutes bioactive forms like α-tocopherol, β-tocopherol, γ-tocopherol, and δ-tocopherol, showed antioxidant properties, which in turn prevent β-amyloid generated due to oxidative stress [266]. It also inhibits cyclo-oxygenase, inhibits secretase enzymes, and protects against tau protein aggregation. This signifies that a decrease in serum vitamin E levels increases the risk of AD development. A clinical trial with Fortasyn Connect (Souvenaid), a multi-nutrient supplement that contains active constituents like eicosapentaenoic acid, choline, uridine monophosphate, and vitamins, improved conditions like cognition and reduced brain atrophy in patients with AD [267].

**Figure 4:**
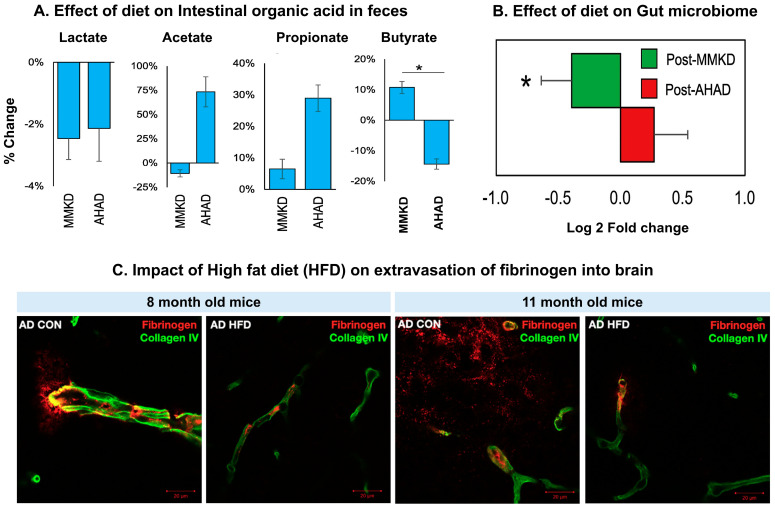
(**A**) Randomized, cross-over, double-blind study including comparison of the Mediterranean ketogenic diet (MMKD) and the American Heart Association Diet in 17 subjects aged 64.6 ± 6.4 years. MMKD reduced fecal lactate and acetate while increasing propionate and butyrate. Conversely, the AHAD increased fecal acetate and propionate while reducing butyrate (* *p* < 0.05) [269]. (**B**) Effect of MMKD and AHAD on gut microbiome and SCFA association with Alzheimer’s biomarker (* *p* < 0.05). (**C**) HFD-fed mice showed less fibrinogen extravasation and Aβ deposition in the brain and improved cognitive function. AD-CON mice showed extensive fibrinogen (red) extravasation from blood into the brain compared to AD-HFD mice [270].

Although there is no effective cure for dementia, some dietary interventions have helped to slow its progression. Diets rich in saturated fatty acids can promote the progression of dementia [258]. Specific diets such as MAD, MIND, Mediterranean, and Modified Atkins are rich in fibers and antioxidants, which protect against neurodegenerative processes. Compared to other diets, the MIND diets show a lower risk of developing AD. Curcumin, folic acid, caffeine, aloe vera, and pomegranate exert beneficial effects on the course of AD disease. Various nutritional supplements, along with a healthy, balanced diet, reduced the progression. Supplements like Anatabloc (R) are a mint lozenge that affects blood levels of β-amyloid (NCT01669876). Rebuilder is another botanical dietary supplement used for subjects with mild to moderate AD (NCT03611439). NT-020 contains vitamin D3, grape extract, green tea extract, and wild blueberries (NCT01963767). Souvenaid is another beverage containing a mixture of vitamins, uridine monophosphate, and omega-3 fatty acids (NCT04147624). Tahini is an oily paste obtained from sesame seeds with natural health-promoting benefits and helps in managing oxidative stress (NCT04608747). NeuroQ is a dietary supplement that includes phosphatidylserine, curcumin (from Turmeric), coffee fruit, ginkgo, propolis, and gotu kola (Brahmi) in a veggie capsule to improve cognitive function (NCT04149639). Magnetin is a magnesium L-Threonate (NCT02210286), and NIC5-15 (or Pinitol), a naturally occurring small molecule [268] found in foods (e.g., pine bark) and medicinal plants (NCT00470418, NCT01928420) are being used for AD. Figure 4 presents studies on diet in AD. In a randomized, crossover, double-blind study comparing a Mediterranean ketogenic diet (MMKD) with the American Heart Association Diet, 17 subjects aged 64.6 ± 6.4 years were assessed. The MMKD reduced fecal acetate and lactate (Figure 4A) while increasing butyrate and propionate. Such findings encourage exploring microbiome- and nutrition-related biomarkers, which could reduce AD risk. Table S1 lists clinical studies on dietary foods and supplements for AD.

**Table 3:** List of studies showing diets for AD management with potential active compounds and their effects.

Culinary Medicine	Mechanism	Active Chemical	Outcome/Result/Efficacy	Ref.
Mediterranean diet containing 40 mL of coconut oil (20 mL during breakfast and lunch each) for only 21 consecutive days	Medium-chain triglycerides are metabolized to produce ketone bodies, which exerts neuroprotective effects.	Medium-chain triglyceride containing lauric acid, caprylic acid, and linolenic acid.	Effective on temporal orientation, visuospatial memory, and semantic memory.	[271]
High glycemic diet containing potatoes, starchy vegetables, refined grains, whole grains, lean and fatty meats, and added sugar (negative effect)	Impaired glucose metabolism, insulin resistance, and type 2 diabetes are factors for amyloid deposition in the brain.	Sugar and carbohydrate.	In cognitively normal adults, a high-glycemic diet raises global and regional cerebral amyloid burden.	[272]
Modified Atkins contains fat (large amount), carbohydrates (low amount), and protein (moderate).	Promote metabolic ketosis.	Carbohydrates, fat, and proteins.	Enhances cognition, episodic memory, and mood Improves liveliness in patients with AD.	[260]
Supplement containing probiotics and selenium: selenium (200 μg/day) with a probiotic (2 × 10^9^ CFU/day each) for 12 weeks.	Reduces tau protein, enhances gut bacterial composition, enhances blood levels of GSH and TAC, and decreases hs-CRP protein levels.	Probiotics containing *Lactobacillus acidophilus*, *Bifidobacterium bifidum*, and *Bifidobacterium longum.*	Significant increase in cognitive function.	[250]
Vitamin E supplementation.	Suppresses cyclooxygenase, associates with antioxidant and anti-inflammatory activity, and inhibits secretase, which is responsible for amyloid production.	Alpha, beta, and gamma tocopherols.	Meta-analyses indicate that individuals with low serum levels of vitamin E are prone to the development of AD.	[266]
Lipid-based diet containing Souvenaid (125 mL per day).	Neuroprotective.	DHA, eicosapentaenoic acid, choline, uridine monophosphate, vitamin B12, B6, C, and E, folic acid, phospholipids, and selenium.	Improves cognition, reduces brain atrophy, and slows disease progression in AD.	[267]
Mediterranean diet containing extra virgin olive 20–30 g per day.	Neuroprotective.	Extra virgin olive oil polyphenols (280 ppm).	Individuals who adhere to a Mediterranean diet plus extra virgin olive oil increase scores of short-term improvements than those following the med diet alone.	[273]
Modified ketogenic diet containing green vegetables, meats, eggs, nuts, seeds, creams, and natural oils.	Promotes physiological ketosis.	58% Fat (26% saturated, 32% non-saturated), 29% protein, 7% fiber, and 6% net carbohydrate by weight.	Compared with a regular diet, patients adhered to a ketogenic diet improves daily function and quality of life in hospitalized patients with AD.	[261]
Mediterranean diet containing Walnut oil.	Enhances mitochondrial function and reduces β-amyloid (1–40).	Linoleic acid, oleic acid, α-linolenic acid, and gamma and beta tocopherol.	Raises ATP production, increases neuronal function, and decreases AD.	[262]
MIND diet containing green leafy vegetables, nuts, berries, beans/legumes, whole grains, fish, poultry, olive oil, and wine.	Anti-inflammatory and antioxidant effects.	Nutrients like folate, lutein-zeaxanthin, vitamin E, and flavonoids.	Improves cognition function and brain plasticity.	[263]

### 4.3. Multiple Sclerosis

**Figure 5:**
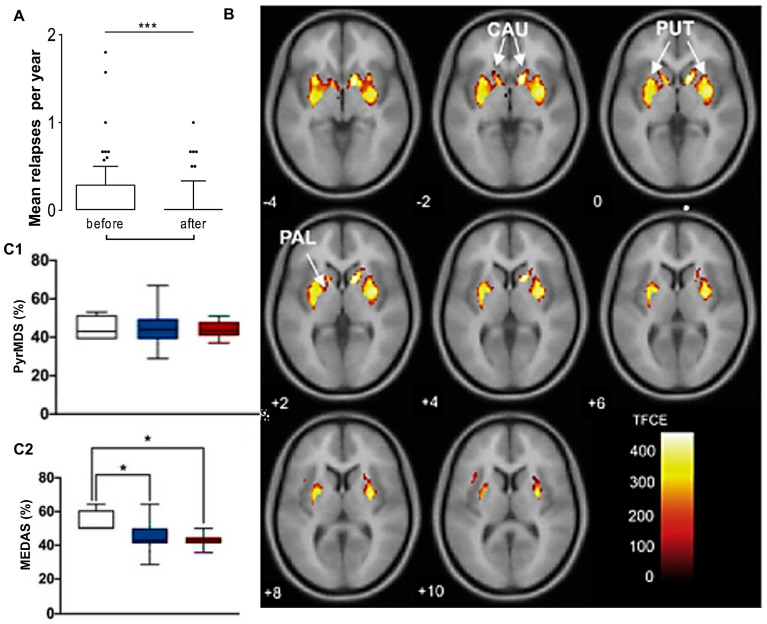
Impact of diet on patients with multiple sclerosis. (**A**) Long-term supplementation with propionic acid (PA) in 97 patients with MS, where patients were stratified with ARR (annual relapse rate) [304]. Further, it was concluded that 41.2% patients showed improvement while 47.4% remained stable, and 11.3% exhibited increased ARR after PA supplementation (*** *p* < 0.001). (**B**) MRI scans of twenty-two patients supplemented with PA. The results concluded a significant reduction in global and regional volumes (* *p* < 0.05) (**C1**,**C2**). Evaluated adherence to the Mediterranean diet in patients with multiple sclerosis [305]. This was assessed using MSSS (MS severity score), MEDAS (Mediterranean Diet Adherence Screener), and PyrMDS (Pyramid-based Mediterranean diet score). Further, pwMS (people with multiple sclerosis) was divided by MSSS: Mild (0–1.7), Intermediate (1.7–5), and Aggressive (>5).

MS is a relapsing and chronic inflammatory disease. It is one of the leading causes of neurological disability [274,275]. It is characterized by CNS lesions and myelin destruction, resulting in severe neurological, cognitive, and physical disabilities. The disease shows two pathological hallmarks: demyelination with inflammation and proliferation of astroglia (gliosis) and degeneration [276]. This interrupts communication between the brain and the rest of the body [277]. Signs and symptoms include numbness or weakness in one or more limbs, the sensation of electric shock, wholly or partially loss of vision, slurred speech, dizziness, and fatigue [274]. This disease is characterized by multifactorial, heterogeneous, and immune-mediated disorder influenced by genetic and environmental factors [278]. Risk factors include low vitamin D levels [279], smoking [280], and insufficient sun exposure [281]. Effective dietary strategies can help reduce courses and improve function [282]. Current therapies for MS effectively reduce relapses, reduce new lesions, and aim at remyelination [283]. Dietary factors can dampen inflammation, prevent demyelination, and combat oxidative stress, which helps to restore functionality [284]. Diet plays a significant role in maintaining cholesterol levels, body weight, and other vascular factors directly affecting MS [285,286]. Further, food with caloric restriction has been shown to improve neuronal injury. The impact of diet on patients with MS is presented in Figure 5. Consumption of an isocaloric diet containing coconut oil and Epigallocatechin Gallate provides anti-inflammatory and antioxidant benefits, offers neuroprotective, and reduces MS-related anxiety levels [287]. Dietary supplements like omega-3 fatty acids, vitamin A, vitamin D, vitamin D3, coenzyme Q10, lipoic acid, and many more exert anti-inflammatory and antioxidant effects. Coconut oil is rich in medium-chain fatty acids like lauric acid and stearic acid, which shows neuroprotective ability. Epigallocatechin acts as an antioxidant and anti-inflammatory agent [288], which reduces disease-related anxiety, inflammation, and disability. PUFAs are found in fish, flax seeds, and walnuts, and they help reduce inflammation through conversion to Prostaglandin E1 and Prostaglandin E2. PUFAs also prevent demyelination and promote neuroprotection [289]. Polysaccharide-based dietary supplements contain bioactive compounds such as polysaccharides, vitamins, and antioxidants exerts immunomodulatory effects and upregulates IL-2, TNF-α, and CD95+ but decreases IL-1β [290]. Polysaccharide-based dietary supplementation can potentially reduce MS-associated infection after 12 months of diet consumption [291]. A high-fat diet is linked to upregulation of IL-6, IL-1β, and Th17. This induces obesity and inflammation (via pro-inflammatory mediators and induction of gut permeability) and leads to MS development [292]. Other than specific dietary components, specific diets like the Paleolithic diet, Mediterranean diet, and MIND diet are beneficial in MS. The MIND diet provides neuroprotection, promotes remyelination of neurons, and acts as disease modifier [293]. A prospective study of pediatric MS included 219 individuals to investigate the link between the energy intake from a fat source and the rate of relapse [294]. The intake of saturated fats in children with MS was associated with an increased risk of relapse. Dietary metabolites from food and changes in gut microbial composition significantly affect MS through G-protein-coupled receptor signaling and inhibition of HDAC (histone deacetylases) [295]. Both these signaling pathways are associated with anti-inflammatory effects. Increased intake of saturated fats increases low-density lipoprotein and cholesterol levels, which are linked to poor outcomes in MS [296]. Saturated fats directly impact the immune system by activating proinflammatory factors like toll-like receptors and down-streaming NF-kβ pathway [297]. Table 4 summarizes the role of diet interventions in MS.

**Table 4:** List of studies showing diets for MS management with potential active compounds and their effects.

Culinary Medicine	Mechanism	Active Chemical	Outcome/Result/Efficacy	Ref.
Mediterranean diet containing Epigallocatechin gallate (EGCG) and coconut oil (800 mg of EGCG, 60 mL of coconut oil).	Neuroprotective antioxidant, anti-inflammatory agent, and anxiolytic agent.	Coconut oil: palmitic acid, myristic acid, lauric acid, stearic acid, and oleic acid; Epigallocatechin gallate (EGCG): polyphenol.	Improves state anxiety and functional capacity; decreases IL-6.	[287]
Polysaccharide-based multi-nutrient dietary supplements (3 times per day).	Increases IL-2, TNF-α, EGF, CD95+, andCD34+ but decreases IL-1b.	Polysaccharides, antioxidants, phytochemicals, vitamins, and minerals.	Improves immune functions and reduces infection rates.	[291]
Modified Paleolithic diet (Two servings per week).	Paleolithic diet increased key nutrients targeting brain function including omega-3-fatty acids, vitamin B, antioxidants, and coenzyme Q10.	Modified Paleolithic diet-contains fibers, potassium, and essential fatty acids.	Modified Paleolithic diet induces clinical improvements in patients with MS.	[259]
Medium-chain triglyceride (MCT)-based ketogenic diet containing coconut oil.	Improved mitochondrial function; decreased oxidative stress.	High fat and low carbohydrate.	MCT-based ketogenic diet induces ketosis but does not induce clinical improvement.	[298,299]
MIND diet containing fruits, vegetables, legumes, nuts, whole grains, dairy, fried foods, processed meats, and fat intake (Servings/day).	Neuroprotection, encourages remyelination and acts as a disease modifier.	High intake of omega-3-fatty acids and polyunsaturated fatty acids.	MIND diet score is linked to thalamic volume; individuals in the highest quartile of MIND diet scores had more significant thalamic volumes versus those in the lowest quartile. For individual food/nutrients, higher intakes of full-fat dairy were associated with lower T2 lesion volumes. Higher intakes of marine omega-3 fatty acids were associated with greater NAWM microstructural integrity.	[293]
Protein source containing red meat (g/week), poultry (serves/week), fish (serves/week), shrimp, and eggs; fruit and vegetable (g/day); butter; and dietary supplements, multi-vitamins, calcium, fish oil, iron, folic acid, protein, vegetable, and dairy products.	Fruits and vegetables have anti-inflammatory properties; red meat causes inflammation.	-------	Subjects with a higher diet-related inflammatory index has a higher risk for MS onset compared with those adhering to a more anti-inflammatory diet regimen. Improving nutritional a pattern through educational programs is likely to reduce MS risk.	[300]
High-fat diet (HFD diet) (Negative effect).	HFD diet causes the production of proinflammatory cytokines, IL-6, IL-1β, and Th17, which causes gut inflammation and CNS autoimmunity, finally leading to MS.	45 kcal% Fat and dextrose.	HFD diet induces obesity, leading to the production of pathogenic bacteria, which can induce inflammation via the induction of gut permeability and pro-inflammatory mediators.	[292]
Mediterranean diet.	Improves lipid profile, modulates inflammation, and increases antioxidant property.	Fruits, vegetables, unprocessed cereals, unsaturated fats, and wine (300 mL/day)	Significant effects on MS are probably mediated by modulation of the gut microbiota and low-grade chronic systemic inflammation.	[301]
Calorie restriction (CR) diets.	Decreases body fat and weight; decreases oxidative stress and inflammation.	Polysaccharide-based multinutrient formula.	CR diets are beneficial for achieving weight loss and modulating emotional health.	[291,302]
Modified Paleolithic elimination (Wahls) diet (4 servings); low-saturated-fat (Swank) diets (6–9 servings).	Modifies neuroinflammation and oxidative stress; boosts mass and diversity of gut microbiota.	5 g of cod liver oil and vegetable oil or fish oil (10–15 g).	Both diets significantly reduced fatigue and improved quality of life in patients with relapsing-remitting MS.	[232]
Calorie restriction (CR) diets (5 days per week).	Anti-inflammatory and neuroprotective.	Carbohydrate, protein, and fat.	CR diets are safe and effective ways to achieve weight loss in people with MS, altering the circulating metabolome and cell subsets, but without change in adipokine levels.	[303]

Table S1 includes a list of clinical studies associated with various culinary foods and supplements in MS. This also represents the data based on different age groups and clinical phases (1–4). Some special supplements include NBT-NM108, a high-fiber dietary formula that helps improve gut function (NCT04574024). Some specific diets, including the MIND diet, Mediterranean diet, Swank Diet, Paleo diet, Wahls Elimination Diet, ketogenic diet, and Gluten-free diet, have been shown to improve patient’s overall health. MIND has emphasized nutrients associated with preventing dementia [223]. The Wahls diet includes the elimination of specific dietary antigens, including gluten, casein, and increased micronutrient content. In contrast, the Swank diet includes a low-saturated-fat diet that prevents disease progression and improves the quality of life. 

### 4.4. Amyotrophic Lateral Sclerosis

ALS is a neurodegenerative disorder characterized by muscular paralysis associated with neurodegeneration [306]. It includes the loss of upper and lower motor neurons in the nuclei of the brain stem, motor cortex, and anterior horn of the spinal cord [307]. It primarily affects the motor system, and the loss of the extra motor system [308]. ALS is also known as Lou Gehrig’s disease, after it was diagnosed in a baseball player. It often begins with symptoms like limb weakness, muscle twitching, and slurred speech [309]. These symptoms progresses to problems in walking, difficulty swallowing, and cognitive and behavioral changes [310,311]. Although ALS’s genetic and environmental roots are still unclear, dietary linkage to oxidative stress has been addressed [312]. Diets with high-fat and glutamate content correlates with a much higher risk of ALS than a diet based on phytochemicals with antioxidants and anti-inflammatory properties [313]. Table 5 summarizes the studies on diets beneficial for ALS.

A clinical study investigated the association between the intake of essential nutrients and ALS. Vitamin E exerts antioxidant effects [protecting against ROS and reactive nitrogen species (RNS)], and vitamin D exerts a neuroprotectant effect to prevent ALS. In contrast, the intake of essential fatty acids like linolenic acid increases oxidative stress (TNF-α induces apoptosis) and inflammation, contributing to ALS. Thus, it shows that linoleic acid is associated with a risk factor for ALS [314]. Intake of macronutrients like carbohydrates has also been linked to disease progression due to genetic predisposition and shortage of the insulin mitochondrial axis. Therefore, individuals associated with ALS should avoid consuming carbohydrates [315]. Medium-chain triglycerides from coconut oil provide ketone bodies, which help address mitochondrial malfunction and impaired energy production in patients with ALS. Coconut oil acts as a neuroprotectant, which improves motor function and survival, and can be used as a prophylactic treatment for ALS [316].

Diets based on anti-inflammatory and antioxidant compounds reduce the risk of ALS, which includes curcumin, vitamin A, vitamin E, vitamin C, coenzyme Q10, and creatine. Spirit 1 is a food supplement used to treat ALS, a combination of phospholipids and antioxidant medicinal plants (NCT02588807). Specific diets, including the American Association of Retired Persons (AARP) and the Paleolithic diet, ease the symptoms associated with ALS (NCT00377351). This diet includes minimally processed food and excludes grains, dairy, and legumes. Hirsutella Sinensis Nutrient Supplements (NCT05284149 and NCT05284149) are given for ALS and Huntington’s disease. Dietary supplements like PolyMVA (NCT04557410), a combination of amino acids, vitamins, and minerals, are used by patients to improve their quality of life. The main active component of this supplement is Palladium lipoic acid complex. Table S4 lists clinical studies associated with dietary foods and supplements for ALS, Huntington’s disease, and spinal muscular atrophy.

**Table 5:** List of studies showing diets for ALS management with potential active compounds and their effects.

Culinary Medicine	Mechanism of Action	Bioactive Compounds/Chemical Constitutes	Outcome/Results/Efficacy	Ref.
Essential vitamins containing vitamin D and vitamin-E.	Neuroprotective and antioxidant effects.	25-OHD and tocopherols.	Vitamin D and vitamin E are defensive in the case of ALS.	[314]
High-caloric fatty diet (HCFD) (405 kcal/day).	Stabilizes body weight.	30mL of HCFD three times a day.	Diet intake decreases weight loss and increases survival of individuals with ALS.	[317]
Coconut oil.	Neuroprotective.	Medium-chain triglycerides.	Retards disease symptoms, increases motor function, increases survival, and stabilizes body weight in mice.	[316]

### 4.5. Huntington’s Disease

It is an inherited disease that causes progressive degeneration of neuronal cells in the brain. It usually affects people between 30 and 40 years. However, if it occurs before 20 years of age, it is called juvenile Huntington’s disease [318]. It arises due to the mutation in the Huntington gene (HTT) caused by the repetition of the CAG unit [319]. It involves complex pathophysiology, like the mutant gene, which disrupts immune and mitochondrial function and transcription [320]. Signs and symptoms include movement disorders (involuntary jerking, muscle problems, slow eye movement, impaired walking, posture, and balance), cognitive disorders (learning difficulty, lack of awareness, and focus), and psychiatric disorders (sadness, social withdrawal, insomnia, and fatigue) [320]. Patients typically die from lack of nourishment, dysphagia, improper swallowing of food, and fatal choking [321].

**Figure 6:**
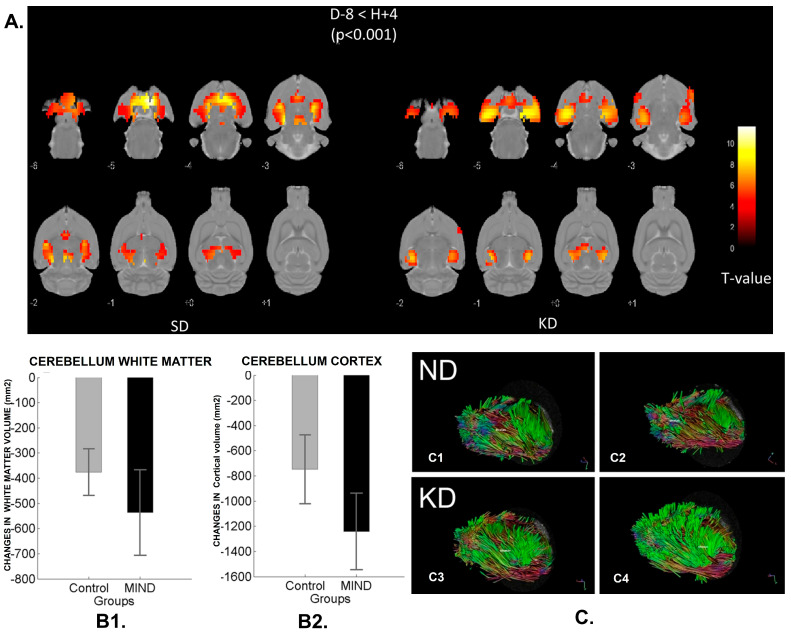
Role of diet in overall brain health. (**A**) PET images of rats with induction of status epilepticus on a one-week ketogenic diet (KD) and its impact on brain glucose metabolism changes [325]. Both groups fed with a standard diet (SD) and KD exhibit equivalent increase in brain metabolism within the same areas of the brain. A 3-month randomized study focusing on the impact of the MIND diet on cognitive performance and changes in brain volume [217]. (**B1**,**B2**) Reduced cerebellum white matter and gray matter are observed more in the MIND group than in the control group. (**C**) Tractograms showing the distribution of fibers correlated with specific brain regions in 20 male Wistar rats fed with a normal diet (**C1**,**C2**) or KD (**C3**,**C4**) [326].

Individuals at risk of developing HD are typically advised to incorporate specific foods and supplements rich in antioxidants and healthy fats into their diet. However, a lower intake of dairy products can delay the onset of this disease [311]. Various phytochemicals, including curcumin, rutin [322], and polyphenols, exhibit anti-inflammatory, antioxidant, and neuroprotective properties that help prevent HD [323]. Epigallocatechin-3-gallate is a primary polyphenolic compound in green tea that exerts an antioxidant effect, reduces striatal damage, and decreases mutant huntingtin-induced neurodegeneration in Drosophila [47]. Diets containing olive oil, fish oil, etc., provide PUFAs and monounsaturated fatty acids (MUFAs), which maintain muscle tone by increasing GABA levels, thereby decreasing unwanted weight loss associated with HD [324]. Table 6 summarizes studies on diet for HD. The clinical status of specific diets and other dietary supplements for HD is summarized in Table S4. Figure 6 presents some important studies showing the diets that promote overall brain health in HD.

### 4.6. Other Neurodegenerative Conditions

Spinal muscular atrophy is a neuromuscular disorder characterized by a defect in the SMN1 gene, which destroys alpha neurons [327]. While Friedrich’s ataxia is an autosomal recessive disorder, it is the most common hereditary ataxia, with 3–4 cases per 100,000 individuals [328]. This disease includes limb ataxia, dysarthria, lower limb areflexia, and decreased leg muscle strength [329]. Other non-neurological complications include diabetes mellitus and hypertrophic cardiomyopathy. It is associated with GAA trinucleotide expansion in the first intron of the frataxin gene [330]. Frataxin is a mitochondrial protein [331], and its deficiency leads to ROS generation [332]. The use of histone deacetylase inhibitors and other pathways upregulating the frataxin is a promising approach to treating this disease [333]. Other than Friedrich’s ataxia, spinal muscular atrophy, dementia associated with Lewy bodies, and PD are common neurodegenerative diseases. Spinal muscular atrophy is another autosomal recessive genetic disorder characterized by progressive muscular atrophy. It is a motor neuron disease resulting from a mutation in the SMN1 gene [334]. Patients affected with Lewy bodies show cognitive impairment, neuropsychiatric symptoms, sleep disturbances, and motor dysfunction [335]. Patients suffering from spinal muscular atrophy showed increased dodecanoic fatty acids in plasma [336].

Due to the deteriorating impacts of such disorders, managing health and wellness is very crucial. Nutrition is of primary importance in such neurodegenerative diseases. Various studies have reported that supplements like Oxepa, Jevity, and long-term adherence to a high-fat diet in patients with ALS can increase survival by 38% (NCT00983983). Using antioxidants and dietary supplements, including coenzyme Q10, vitamin E, selenium, and idebenone, has shown benefits in Friedrich’s ataxia [337,338]. Table S4 summarizes clinical studies on specific diets for spinal muscular atrophy.

## 5. Future Perspective and Conclusions

Functional foods have emerged as a potential strategy in preventing and treating neurodegenerative diseases. Several studies have shown that neuronal dysfunction occurs due to the release of pro-inflammatory mediators, which can be prevented and treated with micronutrients. However, the dose of micronutrients should be tuned to get the desired pharmacological effects. Most of these micronutrients restore normal body function and replenish the body’s deficiencies. However, precise monitoring of food intake, microbiome studies, and mapping of genetic variants requires further investigation. The area of nutrigenomics is evolving and could be a focus for future studies. Future studies could consider studying the health-promoting genes and reducing the expression of disease-promoting genes [339]. This could generate new avenues for precision nutrition and dosing. Additionally, the appendix’s role in immune system modulation, as a store of microbiomes and brain functions, is emerging but is minimally explored. Further research is needed to understand the relationship between the appendix, gut health, and brain health.

In conclusion, adopting a balanced and nutrient-rich diet, along with supplementation of essential nutrients and the inclusion of bioactive phytochemicals, can play a crucial role in reducing the risk and managing neurodegenerative diseases. Ongoing research and clinical trials continue to highlight the importance of dietary interventions as promising alternatives for promoting brain health and preventing cognitive decline.

## Figures and Tables

**Table 1:** List of mitophagy inducers impacting mitochondrial and brain function.

Mitophagy Inducer	Source	Impact on Mitochondrial Function	Impact on Brain Function
Urolithin A	Gut microbiota–derived metabolite (natural polyphenol ellagic acid)	Activation of mitophagy [71]	Reduces neuroinflammation and improves cognition [89]
Resveratrol	Non-flavonoid polyphenol	Relieves the oxidative stress, reduces mitochondrial damage, and prevents apoptosis [81]	Mitophagy imparts neuroprotective effects [90]
Palmatine	Alkaloid from *Coptis chinensis* and *Berberis aristata*	Enhances mitochondrial membrane potential and reduces mitochondrial ROS. [81]	Improves cognitive function in an AD mouse model [91]
Tetrahydro-cannabinol	Cannabinoid from *Cannabis sativa*	Activates mitophagy via the PINK1/Parkin pathway [92]	Modulates neuronal energy metabolism [93]
Berberine	Alkaloid from *Coptis chinensis* and *Berberis aristata*	Activation of the AMPK pathway that induced mitophagy [94]	Alleviates cognitive dysfunction, imparts neuroprotection, and inhibits Aβ production [95]
Quinic acid	Polyphenol from millet	Activates mitochondrial ATP synthase–dependent respiration [96]	Reduces high-fat diet–induced brain oxidative stress [85]
Catechin	Flavonoid from green tea	Promotes mitochondrial biogenesis [96]	Prevents neurodegeneration [97]
Curcumin	Polyphenol from *Curcuma longa*	Induction of mitophagy [98]	Alleviates mitochondrial dysfunction in the brain [99]
Astaxanthin	Carotenoid from microglia	Induction of mitophagy through PINK1/PARKIN pathway activation [98]	Reduces Aβ production [100]
Spermidine	Polyamine obtained from putrescine	Induction of mitophagy through PINK1/PARKIN pathway and improvement of mitochondrial respiration [101]	Reduces Aβ levels and improves cognitive function [101]

**Table 6:** Role of food as medicine/diet in Huntington’s disease treatment.

Food as Medicine	Mechanism of Action	Bioactive Compounds/Chemical Constitutes	Outcome	Ref.
Curcumin.	Prohibits gastrointestinal intestinal dysfunction, conserves homeostasis of the intestine, and decreases malabsorption.	Polyphenols.	Normal motor function, preserved neuropathology, GI upset, GI emptying, and intestinal contractility.	[323]
Rutin (25 and 50 mg/kg).	Antioxidant activity.	Flavonoids.	For Huntington’s treatment, rutin may be a drug of choice.	[322]
Green tea.	Decreases mutant Huntingtin-associated neurodegeneration in Drosophila.	(−)-Epigallocatechin-3-gallate.	Green tea consumption indicates positive effects on symptoms of Huntington’s disease.	[47]
Diet containing olive oil and fish oil.	Increases GABA levels.	Polyunsaturated fatty acid (PUFA) and monounsaturated fatty acid (MUFA).	Maintains muscle strength and decreases unwanted weight loss associated with Huntington’s disease.	[324]
Diet containing fruits and vegetables	Increases antioxidant levels and promotes neurogenesis.	Polyphenols and flavonoids.	Improves anxiety and decreases depression.	

## Supplementary Materials

The following supporting information can be downloaded at https://www.mdpi.com/article/10.3390/antiox14091078/s1; Tables S1–S4 present various clinical trials based on different neurodegenerative diseases.

## Abbreviations

AD: Alzheimer’s disease; ANS: Autonomous nervous system; ALS: Amyotrophic lateral sclerosis; ATP: Adenosine triphosphate; AMPK: AMP-activated protein kinase; Aquaporin-4: (AQP4); BBB: Blood–brain barrier; BDNF: Brain derived neurotrophic factor; CNS: Central nervous system; CR: Caloric restriction; CRM: Calorie restriction mimetics; COX1: Cyclooxygenase 1; DALYS: Disability adjusted life years; DASH: Dietary approaches to stop hypertension; DHA: Docosahexaenoic acid; ENS: Enteric nervous system; GSK3β: Glycogen synthase kinase 3β; GPCR: G-protein coupled receptor; HD: Huntington’s disease; IL-6: Interleukin 6; LPS: Lipopolysaccharide; MS: Multiple sclerosis; MIND: Mediterranean diet; MAD: Modified Atkins diet; MUFA: Monounsaturated fatty acids; MCT: Medium chain triglyceride; mPTP: mitochondrial permeability transition pore; NRF: Nuclear respiratory factor; NF-kB: Nuclear factor-kB; PD: Parkinson’s disease; PGC-1α: Peroxisome proliferator-activated receptor gamma coactivator-1α; PUFA: Polyunsaturated fatty acids; PPAR: Peroxisome proliferator activated receptors; PINK1: PTEN-induced kinase 1; RNS: Reactive nitrogen species; ROS: Reactive oxygen species; SCFA: Short-chain fatty acids; SMN1: Survival motor neuron 1; TNF: Tumor necrosis factor; ULK1: UNC-51 like kinase 1; VNS: Vagus nerve stimulation.
