# Supplementary material for: Management and Prevention of Neurodegenerative Disorders: Can Antioxidant-Rich Dietary Interventions Help?
Source: Antioxidants (Basel). 2025 Sep 2;14(9):1078. doi: 10.3390/antiox14091078 (PMC12466820; doi:10.3390/antiox14091078)
Supplement: Supplementary file 1 [file antioxidants-14-01078-s001.zip › antioxidants-3785137-supplementary.pdf]

Supplementary Information

Table S 1. Clinical trial data of dietary interventions associated with multiple sclerosis

Phases & Status	Interventions	Sponsor/Collaborators	Age (Years)	Enrollment	Study Type	Locations	NCT Number
Phase 4 (C)	Fish Oil	CoordinaciÃ³n de InvestigaciÃ³n en Salud, Mexico	18-55	50	Interventional	Mexico	NCT01842191
Phase 4 (UN)	Vitamin A	Tehran University of Medical Sciences	20-45	20	Interventional	Islamic Republic of	NCT01705457
Phase 4 (C)	Vitamin A	Tehran University of Medical Sciences	20-45	36	Interventional	Islamic Republic of	NCT01225289
Phase 4 (UN)	Vitamin A	Tehran University of Medical Sciences	20-45	100	Interventional	Islamic Republic of	NCT01417273
Phase 4 (NR)	25(OH)D3; vitamin D3	Tehran University of Medical Sciences; Boston University	18-55	54	Interventional		NCT05340985
Phase 4 (T)	Vitamin D3	Carmel Medical Center	18-65	45	Interventional	Israel	NCT01005095
Phase 4 (C)	Vitamin D3; calcium carbonate	University Hospital of North Norway	18-50	80	Interventional	Norway	NCT00785473
Phase 4 (C)	Vitamin D	University Medical Centre Maribor; Medical Faculty Maribor	18-60	89	Interventional	Slovenia	NCT03385356
Phase 3 (T)	Zoledronic Acid; Calcium and Vitamin D combination	Novartis	18-75	29	Interventional	Germany	NCT01166178
Phase 3 (C)	Vitamin D3	Medical University of South Carolina; Thrasher Research Fund	16-45	564	Interventional	United States	NCT00412087
Phase 3 (UN)	Vitamin D	Mazandaran University of Medical Sciences	18-50	240	Interventional	Islamic Republic of	NCT01768039
Phase 3 (C)	Vitamin D3	Johns Hopkins University; Oregon Health and Science University;	18-50	172	Interventional	United States	NCT01490502

		University of California, San Francisco; Washington University School of Medicine; Icahn School of Medicine at Mount Sinai; University of Pennsylvania; Yale University; The Cleveland Clinic; University of Rochester; Stanford University; University of Virginia; Swedish Medical Center; Anne Arundel Health System Research Institute; Columbia University; University of Massachusetts, Worcester; Dignity Health					
Phase 2|3 (C)	Fish Oil	Isfahan University of Medical Sciences; Shiraz University of Medical Sciences	18-45	50	Interventional		NCT02939079
Phase 2 (C)	Curcumin	Merck KGaA, Darmstadt, Germany; Merck Serono S.P.A., Italy	18-60	80	Interventional	Italy	NCT01514370
Phase 2 (C)	Coconut oil and epigallocatechin gallate	FundaciÃ³n Universidad CatÃ³lica de Valencia San Vicente MÃ¡rtir; Valencian Institute of Neurorehabilitation Foundation	19-65	60	Interventional	Spain	NCT03740295
Phase 2 (UN)	Vitamin D3	AlJohara M AlQuaiz, M.D.; King Saud University	18-55	200	Interventional	Saudi Arabia	NCT01753375
Phase 2 (C)	Vitamin D3	Merck KGaA, Darmstadt, Germany; Merck Serono S.A.S, France	18-65	129	Interventional	France	NCT01198132
Phase 2 (T)	Vitamin D	Emory University; Dermatology Foundation	18& older	3	Interventional	United States	NCT01498406
Phase 2 (T)	D-aspartate	Merck KGaA, Darmstadt, Germany	18-55	7	Interventional	Italy	NCT03387046

Phase 2 (T)	Polyphenon E	Louisiana State University Health Sciences Center in New Orleans; National Center for Complementary and Integrative Health (NCCIH)	18-60	11	Interventional	United States	NCT01451723
Phase 2 (W)	95% Pure ECGC capsules 200mg	Louisiana State University Health Sciences Center in New Orleans; National Multiple Sclerosis Society	18-65	0	Interventional	United States	NCT02011451
Phase 2 (C)	VigantOL oil plus	Merck KGaA, Darmstadt, Germany	18-55	260	Interventional	Switzerland	NCT01285401
Phase 1|2 (EI)	NBT-NM108 (60 g/day); NBT-NM108 (0 g/day)	Suhayl Dhib-Jalbut, MD; Rutgers, The State University of New Jersey	21-55	50	Interventional	United States	NCT04574024
Phase 1|2 (C)	Vitamin D3	University of Toronto; Direct MS-Proactive Charity; Multiple Sclerosis Society of Canada	18-55	49	Interventional	Canada	NCT00644904
Phase 1|2 (C)	5000IU vitamin D;10000IU vitamin D	University College Dublin; University of Dublin, Trinity College; St Vincent's University Hospital, Ireland	18-55	64	Interventional	Ireland	NCT01728922
Phase 1 (C)	Low fat study diet	Oregon Health and Science University	18-70	61	Interventional	United States	NCT00852722
Phase 1 (C)	Modified paleolithic diet; Omega 3 fatty acids; Maltodextrin fiber supplement; Niacinamide; Methyl B12; Taurine; creatine; thiamine; riboflavin; N acetylcysteine; alpha lipoic acid;	University of Iowa; Direct MS Canada; DJO Incorporated; Pinnaclife Inc.; TZ Press, LLC	18-65	38	Interventional	United States	NCT01381354

	L acetyl carnitine; Coconut oil						
Phase 1 (T)	Linoleic Acid/Oleic Acid	University of Rochester	18-70	9	Interventional	United States	NCT00638196
Phase 1 (UN)	D Mannose	University College, London; UCLH	18-65	20	Interventional	United Kingdom	NCT02490046
Phase 1 (C)	Lipoic Acid	Portland VA Medical Center; Oregon Health and Science University	18& older	69	Interventional	United States	NCT00997438
Phase 1 (C)	Carbidopa; L-Histidine	University of Miami; United States Department of Defense	18-60	18	Interventional	United States	NCT03266965
Phase 1 (C)	Scelectium Tortuosum	Woodbury, Michel, M.D.; PL Thomas & Co., Inc.	45-65	20	Interventional	Puerto Rico	NCT01805518
Phase 1 (C)	Vitamin D3	Johns Hopkins University	18-55	40	Interventional	United States	NCT01024777
Phase 1 (C)	Alpha Lipoic Acid	Rebecca Spain; Oregon Health and Science University	18& older	20	Interventional	United States	NCT03493841
Phase 1 (C)	Lipoic acid (LA); lipoic acid (LA) with fish oil and LA without fish oil	Oregon Health and Science University; National Center for Complementary and Integrative Health (NCCIH)	18-80	40	Interventional	United States	NCT00676156
NA (C)	Omega-3 fatty acids	Icahn School of Medicine at Mount Sinai	18-65	36	Interventional	United States	NCT02986893
NA (C)	Wahls Diet	Terry L. Wahls; University of Iowa	30-65	15	Interventional	United States	NCT01915433
NA (C)	Modified Paleo diet	Amanda Irish; University of Iowa	18-45	31	Interventional	United States	NCT02687919
NA (A, NR)	Swank Diet; Wahls Elimination Diet	Terry L. Wahls; National Multiple Sclerosis Society; University of Iowa	18-70	100	Interventional	United States	NCT02914964
NA (C)	High/Low Sodium Diet	Yale University	18-60	14	Interventional	United States	NCT02282878
NA (C)	Mediterranean Diet	Iaso Maternity Hospital, Athens, Greece; Harokopio University;	18& older	40	Interventional	Greece	NCT05175378

		G.Gennimatas General Hospital					
NA (A, NR)	Ketogenic diet	Charite University, Berlin, Germany	18-65	111	Interventional	Germany	NCT03508414
NA (R)	Ketogenic Diet	Terry L. Wahls; University of Iowa	18-70	156	Interventional	United States	NCT05007483
NA (C)	Ketogenic low glycemic load treatment	Charite University, Berlin, Germany	18-68	48	Interventional	Germany	NCT01538355
NA (R)	Low-fat diet	Oregon Health and Science University; National Multiple Sclerosis Society	18-70	108	Interventional	United States	NCT03322982
NA (C)	DIET-MS	University of Alabama at Birmingham; National Multiple Sclerosis Society	21-65	20	Interventional	United States	NCT03372187
NA (C)	Intermittent fasting	Washington University School of Medicine	18& older	60	Interventional	United States	NCT03539094
NA (UN)	Gluten-free diet	University of Copenhagen; Rigshospitalet, Denmark	18-59	40	Interventional	Denmark	NCT03451955
NA (UN)	Gluten Free Diet	Fondazione IRCCS Ca' Granda, Ospedale Maggiore Policlinico	18-70	40	Interventional	Italy	NCT01867151
NA (R)	High Fiber Diet Intervention	Luxembourg Institute of Health;Centre Hospitalier du Luxembourg; Integrated Biobank of Luxembourg; Luxembourg Centre for Systems Biomedicine	18-55	40	Interventional	Luxembourg	NCT04352231
NA (C)	Caprylic Triglyceride	University of Miami; National Multiple Sclerosis Society; Cerecin	18-59	124	Interventional	United States	NCT01848327
NA (C)	Early Harvest Extra Virgin Olive Oil	Aristotle University Of Thessaloniki; Greek Alzheimer's Association and Related Disorders; Ellis-Farm, Eliama Daily	18-65	30	Interventional	Greece	NCT04120675

		Value Gold (ellis-farm.com)					
NA (C)	Beta-alanine supplementation	Hasselt University	18-75	45	Interventional	Belgium	NCT03418376
NA (C)	Calcium intake	Centre Hospitalier Universitaire de NÄ«mes	18-69	100	Interventional	France	NCT02636829
NA (R)	Lutein	University of Illinois at Urbana-Champaign; Division of Nutritional Sciences, University of Illinois at Urbana-Champaign; National Institutes of Health Rehabilitation Research Resource to Enhance Clinical Trials (REACT)	18-64	60	Interventional	United States	NCT04843813
NA (EI)	N-acetyl Cysteine	Thomas Jefferson University	18& older	55	Interventional	United States	NCT03032601
NA (R)	Prebiotics	Columbia University	18& older	20	Interventional	United States	NCT04038541
NA (NR)	High Phenolic Extra Virgin Olive Oil	University of Cyprus; World Olive Center for Health; Ellis-Farm, Eliama Daily Value	18-65	100	Interventional		NCT04787497
NA (C)	Carnosine, capsulle	University of Novi Sad, Faculty of Sport and Physical Education; CarnoMed	18-65	3	Interventional	Serbia	NCT03995810
NA (C)	Capsules with 160 mg Teavigo (at least 94% EGCG)	Charite University, Berlin, Germany	20-60	20	Interventional	Germany	NCT01417312
NA (C)	Vitamin D3	Johns Hopkins University; University of California, San Francisco; National Multiple Sclerosis Society	18-60	57	Interventional	United States	NCT01667796
NA (C)	Vitamin D3	Maastricht University Medical Center; Orbis Medical Centre	18& older	15	Interventional	Netherlands	NCT00940719

NA (C)	Vitamin D3	Johns Hopkins University; University of California, San Francisco; National Multiple Sclerosis Society	18-60	57	Interventional	United States	NCT01667796
NA (C)	Vitamin D3	Isfahan University of Medical Sciences	23-59	200	Interventional	Islamic Republic of	NCT02696590
NA (C)	Melatonin	University of California, San Francisco	20-70	30	Interventional	United States	NCT04035889
NA (C)	Vitamin D supplementation	University of Surrey; Conselho Nacional de Desenvolvimento CientÃ-fico e TecnolÃ³gico; Universidade Federal de GoiÃ¡s	20-59	136	Interventional	United Kingdom	NCT03318029
NA (C)	Beta-alanine	Hasselt University	18-75	31	Interventional	Belgium	NCT03803800
NA (R)	Ocufolin	University of Miami	18-99	5000	Interventional	United States	NCT03135327
(C)	Modified Atkins diet	University of Virginia	12 to 55	45	Observational	United States	NCT03718247
(A, NR)	Intake of red and processed meat and fibres	University of Southern Denmark; Hospital of Southern Jutland; Odense Patient Data Explorative Network; University of Aarhus; Danish Cancer Society	50-64	57053	Observational	Denmark	NCT03456206
(C)	High sodium diet	FundaciÃ³n para la Lucha contra las Enfermedades NeurolÃ³gicas de la Infancia	18-50	132	Observational	Argentina	NCT01846234

C= Completed; NR= Non-recruiting; A, NR= Active non-recruiting; EI: Enrolling by invitation; T: Terminated; UN: Unknown; NA: Not applicable

Table S 2. Clinical trial data of dietary interventions associated with Parkinson disease.

Phase & Status	Interventions	Sponsor/Collaborators	Age (Years)	Enrollment	NCT Number	Study Type	Locations
Phase 4 (T)	Vitamin D3	Emory University	18-89	31	NCT00571285	Interventional	United States
Phase 4 (C)	Whey protein; Soy protein	Chulalongkorn University	30-80	38	NCT01662414	Interventional	Thailand
Phase 3 (C)	VIUSID/ALZER	Catalysis SL	20-90	100	NCT01016470	Interventional	Cuba
Phase 3 (C)	Probiotics with prebiotic	National University of Malaysia	18 & older	48	NCT04451096	Interventional	Malaysia
Phase 3 (T)	Nutritional supplement creatine	University of Rochester; National Institute of Neurological Disorders and Stroke (NINDS)	Child, Adult, Older Adult	1741	NCT00449865	Interventional	Canada
Phase 2|3 (T)	Vitamin B12 Supplementation	Emory University; National Institutes of Health (NIH)	21 & older	57	NCT00208611	Interventional	United States
Phase 2|3 (C)	coenzyme q10	National University Hospital, Singapore	21 Months and older	20	NCT01892176	Interventional	Singapore
Phase 2|3 (T)	Hydrogen oral tablet	Stony Brook University	40-80	2	NCT03971617	Interventional	United States
Phase 2 (C)	Vitamin D3	Memorial Medical Center; United States Department of Defense	18 & older	23	NCT00907972	Interventional	United States
Phase 2 (C)	Vitamin D3; calcium	VA Office of Research and Development; Oregon Health and Science University	50-99	101	NCT01119131	Interventional	United States
Phase 2 (W)	Coenzyme Q10	National Institute of Mental Health (NIMH); National Institutes of Health Clinical Center (CC)	18-65	0	NCT00327756	Interventional	United States
Phase 2 (R)	Resistant maltodextrin	Northwestern University; University of Illinois at Chicago	60 & older	30	NCT03667404	Interventional	United States
Phase 2 (C)	Metabolic Cofactor	Istanbul Medipol University Hospital; ScandiBio	18 & older	120	NCT04044131	Interventional	Turkey

	Supplementation; Sorbitol	Therapeutics AB; Alanya Alaaddin Keykubat University; Sahlgrenska University Hospital, Sweden; KTH Royal Institute of Technology					
Phase 2 (R)	Probiotic	University of British Columbia; The W. Garfield Weston Foundation	40-80	72	NCT03968133	Interventional	Canada
Phase 2 (UN)	Oral D-Mannitol of Placebo	Hadassah Medical Organization	40-75	60	NCT03823638	Interventional	Israel
Phase 2 (C)	Ubiquinol	Weill Medical College of Cornell University	40-75	11	NCT03061513	Interventional	
Phase 2 (R)	N acetyl cysteine	Thomas Jefferson University	30 & older	50	NCT04459052	Interventional	United States
Phase 2 (C)	Fermented Papaya Preparation; Granulated Sugar	University of Florida; Osato Research Institute	65-100 Years	30	NCT02771366	Interventional	United States
Phase 2 (C)	Green Tea Polyphenols (EGCG/ECG)	Xuanwu Hospital, Beijing; Ministry of Health, China; Michael J. Fox Foundation for Parkinson's Research	30 & older	480	NCT00461942	Interventional	China
Phase 2 (NR)	MitoQ	University of Edinburgh; The Jon Moulton Charity Trust; MitoQ	6 to 17	120	NCT05539625	Interventional	
Phase 2 (NR)	MitoQ	University of Edinburgh; JP Moulton Charitable Foundation; MitoQ	18 & older	206	NCT04276740	Interventional	
Phase 1|2 (C)	Tyrosine Comparator:Sugar Pill	New York Institute of Technology; Michael J. Fox Foundation for Parkinson's Research	50-80	40	NCT01676103	Interventional	United States
Phase 1 (C)	Liquigen MCT oil; Standard American Diet	National Institute of Neurological Disorders and Stroke (NINDS); National Institutes of Health Clinical Center (CC)	50 & older	21	NCT04584346	Interventional	United States

Phase 1 (T)	Modified Atkins diet	Johns Hopkins University	12 to 65	20	NCT00952601	Interventional	United States
Phase 1 (R)	Tributyrin	Nicolaas Bohnen, MD, PhD; University of Michigan	45 & older	20	NCT05446168	Interventional	United States
Phase 1 (C)	Scelectium Tortuosum	Woodbury, Michel, M.D.; PL Thomas & Co., Inc.	45-65	20	NCT01805518	Interventional	Puerto Rico
Phase 1 (R)	High Fiber supplement	Rutgers, The State University of New Jersey	50-75	60	NCT04976959	Interventional	United States
Phase 1 (C)	Docosahexaenoic Acid (DHA)	VA Office of Research and Development; Oregon Health and Science University	21-99	33	NCT01563913	Interventional	United States
Phase 0 (C)	L-tyrosine; Sugar Pill	New York Institute of Technology	40-84	6	NCT02259049	Interventional	United States
NA (UN)	Ketogenic diet	University of Padova	18-100	36	NCT02687698	Interventional	Italy
NA (UN)	Ketone ester drink (carbohydrate containing) drink	University of Oxford	42 & older	20	NCT01364545	Interventional	United Kingdom
NA (NR)	KETOGENIC DIET	Aga Khan University; National Institute of Child Health, Karachi, Pakistan	2 to 15	26	NCT05152771	Interventional	
NA (C)	Ketogenic diet; Healthy high carbohydrate diet	University of Cincinnati	50 & older	62	NCT00777010	Interventional	United States
NA (C)	Ketone Ester Elite endurance Nutrition Drink; Stool Sample	University of Florida	40-75	10	NCT04477161	Interventional	United States
NA (C)	Cocoa	Oxford Brookes University; European Parkinson Therapy Centre	18 & older	30	NCT03288155	Interventional	Italy
NA (UN)	Dark chocolate (85% cocoa); White chocolate (0% cocoa)	Technische UniversitÃ¤t Dresden; University of Wuerzburg	40 & older	30	NCT02275884	Interventional	Germany

NA (UN)	Chocolate	Technische UniversitÃ¤t Dresden	18 & older	23	NCT00906763	Interventional	Germany
NA (R)	Lactobacillus casei DG (Enterolactis duoÂ®)	University of Salerno; Roberto Erro; Maria Teresa Pellecchia; Antonella Santonicola; Carolina Ciacci	18-75	30	NCT04293159	Interventional	Italy
NA (C)	Mediterranean Diet	University of Florida	21-85	8	NCT03851861	Interventional	United States
NA (NR)	Mediterranean diet supplemented with medium-chain triglyceride oil	University of British Columbia; Weston Family Foundation	45-85	50	NCT05469997	Interventional	
NA (C)	Mediterranean diet	University of Florida	40-85	46	NCT04683900	Interventional	United States
NA (T)	Folic Acid, Vitamin B6, Vitamin B12; B6, B12, L-methylfolate; B6, B12, Placebo	Northwell Health	30 & older	150	NCT00853879	Interventional	United States
NA (C)	Coenzyme Q10	Lahey Clinic	30 & older	61	NCT00382824	Interventional	United States
NA (UN)	Low fat western diet; traditional low-fat Western diet; normal diet	Sheba Medical Center; Tel Aviv University	40-80	20	NCT02274324	Interventional	
NA (NR)	Protein Redistribution Diet	University of Alabama at Birmingham	45-99	20	NCT05437640	Interventional	United States
NA (R)	gluten-free diet	General University Hospital, Prague; Czech Academy of Sciences	40-80	90	NCT05238545	Interventional	Czechia
NA (UN)	Protein and calorie-controlled diet	University Hospital, Clermont-Ferrand	18-70	50	NCT01114321	Interventional	France

NA (UN)	Supressi. T-Diet plus Range; High Protein	Vegenat, S.A.	70 & older	184	NCT01192529	Interventional	Spain
NA (C)	Fortiral; Mg++	Ospedale Generale Di Zona Moriggia-Pelascini	60 & older	51	NCT05523791	Interventional	Italy
NA (T)	Protein and calorie controlled diet	University Hospital, Clermont-Ferrand	18-70	8	NCT00663312	Interventional	France
NA (C)	FortifitÂ®	Emanuele Cereda; ASST Gaetano Pini-CTO; U.S. Riabilitazione Parkinson, Fondazione Gaetano e Piera Borghi di Brebbia; Ospedale Generale Di Zona Moriggia-Pelascini; IRCCS Policlinico S. Matteo	Child, Adult, Older Adult	150	NCT03124277	Interventional	Italy
NA (C)	ETHNODYNE VISIO	University Hospital, Strasbourg, France; ETHNODYNE	40-75	24	NCT02815800	Interventional	France
NA (UN)	Prebiotic Bar	Rush University Medical Center	Child, Adult, Older Adult	10	NCT04512599	Interventional	United States
NA (EI)	Probiotics	Franca Marino; UniversitÃ degli Studi del Piemonte Orientale "Amedeo Avogadro"; UniversitÃ degli Studi dell'Insubria	Child, Adult, Older Adult	88	NCT05173701	Interventional	Italy
NA (R)	Hericium erinaceus mycelium	National Cheng-Kung University Hospital	50-79	80	NCT04428983	Interventional	Taiwan
NA (C)	Sarcosine Capsule	China Medical University Hospital; National Science Council, Taiwan	Child, Adult, Older Adult	30	NCT01785628	Interventional	Taiwan
NA (R)	Psyllium; Coarse wheat bran; Maltodextrin	University of Florida	40-85	79	NCT04829760	Interventional	United States

NA (C)	Intravenous and Oral n-acetyl cysteine	Thomas Jefferson University	30-80	51	NCT02445651	Interventional	United States
NA (R)	PS128	Professor Lu Neurological Clinic	20-80	20	NCT04722198	Interventional	Taiwan
NA (C)	PS128	Professor Lu Neurological Clinic	40-80	60	NCT04389762	Interventional	Taiwan
NA (C)	Lactobacillus plantarum PS128	Professor Lu Neurological Clinic	40-80	30	NCT03566589	Interventional	Taiwan
NA (R)	PS128	Professor Lu Neurological Clinic	45-80	120	NCT04722211	Interventional	Taiwan
NA (C)	Nicotine gum	New York Institute of Technology	Child, Adult, Older Adult	10	NCT02452125	Interventional	United States
NA (A, NR)	Multi-strain probiotic	King's College Hospital NHS Trust; King's College London	18 & older	60	NCT05146921	Interventional	United Kingdom
NA (EI)	nano-PSO	Distribuidora Biolife SA de CV	48-65	170	NCT05142085	Interventional	Mexico
NA (R)	Immediate Release Melatonin; Extended Release Melatonin	Mayo Clinic	18 & older	50	NCT05307770	Interventional	United States
NA (C)	Resistant starch; Recommendation with regard to nutrition	UniversitÃ¤t des Saarlandes	18 & older	95	NCT02784145	Interventional	Germany
NA (NR)	Betaquik MCT supplement	UniversitÃ© de Sherbrooke; NestlÃ©	60 & older	20	NCT04322461	Interventional	
NA (C)	High protein	Purdue University	35-69	71	NCT03174769	Interventional	United States
NA (NR)	Beta Hydroxybutyrate Ester	University of Ulm	18 & older	76	NCT04820478	Interventional	Germany
NA (UN)	vitamin B6 (pyridoxine hydrochloride),	New York Institute of Technology; Stony Brook University	50-80	40	NCT01238926	Interventional	United States

	B12 (cyanocobalamin), and Folic Acid supplementation						
NA (UN)	Mind Master	Harokopio University	25-40	62	NCT02837107	Interventional	
NA (UN)	Diary (for reporting all impulse control disorders especially compulsive eating disorders)	University Hospital, Clermont-Ferrand	18-80	60	NCT03818243	Interventional	France
NA (UN)	Carnosine supplementation	Slovak Academy of Sciences; Comenius University; University Hospital Bratislava; National Cheng Kung University	55-80	100	NCT03330470	Interventional	Taiwan

C= Completed; NR= Non-recruiting; A, NR= Active non-recruiting; EI: Enrolling by invitation; T: Terminated; UN: Unknown; NA: Not applicable

Table S 3. Clinical trial data of dietary interventions associated with Alzheimer's disease

Phase & Status	Interventions	Sponsor/Collaborators	Age	Enrollment	NCT Number	Study Type	Locations
Phase 4 (UN)	Curcumin Formulation	Jaslok Hospital and Research Centre; Pharmanza Herbal Pvt Ltd; Verdure Sciences; University of California, Los Angeles	50-80	26	NCT01001637	Interventional	India
Phase 4 (UN)	Curcumin	VA Office of Research and Development	50-90	80	NCT01811381	Interventional	United States
Phase 4 (T)	curcumin + bioperine	Louisiana State University Health Sciences Center Shreveport	55-85	10	NCT00595582	Interventional	United States
Phase 4 (C)	Curcumin	University of California, Los Angeles	50-90	46	NCT01383161	Interventional	United States
Phase 4 (C)	Curcumin C3 Complex	John Douglas French Foundation; Institute for the Study of Aging (ISOA); National Institute on Aging (NIA)	50 & older	33	NCT00099710	Interventional	United States
Phase 4 (C)	Curcum.Luteol.Theaflav.Lip. Acid,FishOil,Quercet.,Resveratr.	Life Extension Foundation Inc.	60-85	12	NCT01716637	Interventional	United States
Phase 4 (C)	Curcumin and ginkgo extract	Chinese University of Hong Kong; BUPA Foundation; Kwong Wah Hospital	50 & older	36	NCT00164749	Interventional	Hong Kong
Phase 4 (NR)	KETOGENIC DIET	Aga Khan University; National Institute of Child Health, Karachi, Pakistan	2 to 15	26	NCT05152771	Interventional	
Phase 3 (UN)	Ketogenic medium chain triglyceride drink	University of British Columbia; UniversitÃ© de Sherbrooke	50-90	40	NCT02912936	Interventional	Canada
Phase 3 (R)	Ketogenic Diet	University of Kansas Medical Center; National Institute on Aging (NIA)	50-90	80	NCT03860792	Interventional	United States
Phase 3 (NR)	Ketogenic diet	Assistance Publique - HÃ´pitaux de Paris; France Alzheimer	50 & older	70	NCT04701957	Interventional	France
Phase 3 (C)	Ketogenic Diet	University of Kansas Medical Center	Child, Adult,	15	NCT03690193	Interventional	

			Older Adult				
Phase 3 (C)	ketogenic drink	UniversitÃ© de Sherbrooke; Alzheimer's Association	55& older	82	NCT02551419	Interventional	Canada
Phase 3 (R)	Omega-3 treatment	University Hospital, Toulouse	70 & older	400	NCT03691519	Interventional	France
Phase 2|3 (C)	omega-3 polyunsaturated fatty acids (EPA+DHA)	Taipei City Psychiatric Center, Taiwan; Department of Health, Executive Yuan, R.O.C. (Taiwan)	55-90	46	NCT00628017	Interventional	Taiwan
Phase 2|3 (C)	omega-3 fatty acids	UniversitÃ© de Sherbrooke	20-35	80	NCT01544855	Interventional	Canada
Phase 2 (NR)	omega-3 phospholipid; omega-3 Triglycerides	UniversitÃ© de Sherbrooke	30-50	160	NCT04279743	Interventional	Canada
Phase 2 (C)	omega-3 fatty acid	UniversitÃ© de Sherbrooke	50-90	40	NCT01577004	Interventional	Canada
Phase 2 (C)	omega-3	University Hospital, Toulouse	70 & older	1680	NCT00672685	Interventional	France
Phase 2 (C)	omega-3 supplementation	Charite University, Berlin, Germany; German Federal Ministry of Education and Research	50-80	330	NCT01219244	Interventional	Germany
Phase 2 (C)	Lipoic Acid plus Omega-3 Fatty Acids	Oregon Health and Science University	55& older	42	NCT01780974	Interventional	United States
Phase 2 (C)	Mediterranean Diet	University of Kansas Medical Center; National Institute on Aging (NIA)	65& older	30	NCT02921672	Interventional	United States
Phase 2 (C)	Mediterranean diet	Pablo PÃ©rez MartÃnez; MaimÃ³nides Biomedical Research Institute of CÃ³rdoba	60 & older	50	NCT05029765	Interventional	Spain
Phase 2 (UN)	Greek Mountain Tea; Mediterranean Diet	Aristotle University Of Thessaloniki	55-85	50	NCT04435509	Interventional	Greece
Phase 2 (UN)	Beverage of Olive Oil Leaves;	Aristotle University Of Thessaloniki	55-85	100	NCT04440020	Interventional	Greece

	Mediterranean Diet						
Phase 2 (R)	Pomegranate oil, Mediterranean Diet	Aristotle University Of Thessaloniki; Greek Alzheimer's Association and Related Disorders	60-85	60	NCT04990362	Interventional	Greece
Phase 2 (A, NR)	Mediterranean Diet; Study Supplement; Low-fat Diet	University of Kansas Medical Center; National Institute on Aging (NIA)	65& older	209	NCT03841539	Interventional	United States
Phase 2 (NR)	Mediterranean diet	University of Salamanca	50 & older	84	NCT04439097	Interventional	Spain
Phase 2 (R)	A Protein Enriched Mediterranean Diet (PROMED) Intervention	Queen's University, Belfast; University College Dublin; Wageningen University; Friedrich-Alexander-UniversitÃ¤t Erlangen-NÃ¼rnberg; Dublin City University	60 & older	105	NCT05166564	Interventional	United Kingdom
Phase 2 (R)	MIND Diet	Rush University Medical Center; University of Chicago; Advocate Hospital System; National Institute on Aging (NIA)	55-80	500	NCT04337255	Interventional	United States
Phase 2 (C)	MIND Diet	Rush University Medical Center; Harvard School of Public Health (HSPH); Brigham and Women's Hospital; National Institute on Aging (NIA)	65-84	604	NCT02817074	Interventional	United States
Phase 2 (W)	MAD; MIND	Johns Hopkins University	60 & older	0	NCT03585907	Interventional	
Phase 2 (R)	MIND foods; Polyphenols as control foods	Indiana University; National Institutes of Health (NIH); National Institute on Aging (NIA)	60 & older	180	NCT03419052	Interventional	United States
Phase 2 (R)	Vitamin D3	University of California, Davis; National Institutes of Health (NIH); National Institute on Aging (NIA)	65-90	180	NCT03613116	Interventional	California, United States
Phase 2 (C)	Vitamine-B and Folic Acid Complex, calcium	Ludwig-Maximilians - University of Munich; European Union; Unilever R&D	4 to 6	250	NCT00811291	Interventional	Germany

Phase 2 (C)	Vitamin D3; gel capsule containing no vitamin D.	University of Dublin, Trinity College	60-80	60	NCT02804841	Interventional	Ireland
Phase 2 (C)	Vitamin B12	Chinese University of Hong Kong	70-100	271	NCT02457507	Interventional	China
Phase 2 (R)	Vitamin D; Vitamin D + L-cysteine; L-cysteine	Louisiana State University Health Sciences Center Shreveport	18-65	120	NCT04939792	Interventional	United States
Phase 2 (R)	Ginkgo Leaf Extract and Armillariella Mellea Powder Oral Solution	Beijing Tiantan Hospital; Beijing Stroke Association	60-85	800	NCT04492241	Interventional	China
Phase 2 (C)	Ginkgo biloba	National Center for Complementary and Integrative Health (NCCIH); Office of Dietary Supplements (ODS); National Institute of Neurological Disorders and Stroke (NINDS); National Institute on Aging (NIA); National Heart, Lung, and Blood Institute (NHLBI)	75& older	3069	NCT00010803	Interventional	United States
Phase 2 (C)	Ginkgo Biloba Extract	Assiut University	20-35	226	NCT02425436	Interventional	
Phase 2 (C)	Aloe Vera with Crocus (saffron); Aloe Vera (simple); Mediterranean Diet	Aristotle University Of Thessaloniki	55-85	100	NCT04436614	Interventional	Greece
Phase 2 (C)	Pomegranate Juice	University of California, Los Angeles; POM Wonderful LLC	50-75	212	NCT02093130	Interventional	United States
Phase 1|2 (C)	POMx	University of California, Los Angeles; POM Wonderful LLC	50-75	212	NCT01950221	Interventional	California, United States
Phase 1|2 (C)	Pomegranate	Dalin Tzu Chi General Hospital	50-80	120	NCT03575390	Interventional	Taiwan

Phase 1|2 (C)	caprylidene	University of California, Los Angeles; John Douglas French Foundation	50-90	17	NCT01122329	Interventional	2United States
Phase 1|2 (C)	Neptune Krill Oil; Fish Oil; (soy oil)	NeuroBioPharm Inc.; Neptune Technologies and Bioressources Inc.	50 & older	175	NCT00867828	Interventional	Canada
Phase 1|2 (A, NR)	Cocoa Extract; Multivitamin	Columbia University; Brigham and Women's Hospital; Fred Hutchinson Cancer Center; Mars, Inc.	60 & older	3947	NCT04582617	Interventional	New York, United States
Phase 1 (W)	Longevinex brand resveratrol supplement	Medical College of Wisconsin	50-90	0	NCT00743743	Interventional	
Phase 1 (UN)	Huperzine A	VA Nebraska Western Iowa Health Care System; American Legion of Iowa Foundation	19-59	15	NCT01012830	Interventional	United States
Phase 1 (UN)	ergothioneine	National University Hospital, Singapore; National University, Singapore; National University Health System, Singapore	60-90	106	NCT03641404	Interventional	Singapore
Phase 1 (T)	Anatabloc(R)	Rock Creek Pharmaceuticals, Inc.; Roskamp Institute Inc.	65-90	85	NCT01669876	Interventional	United States
Phase 1 (R)	Caffeine	University Hospital, Lille; Groupement InterrÃ©gional de Recherche Clinique et d'Innovation; Laboratory of excellence DISTALZ; RÃ©gion Nord-Pas de Calais, France; Meo coffee	50 & older	248	NCT04570085	Interventional	France
Phase 1 (C)	Resveratrol with Glucose, and Malate	US Department of Veterans Affairs; Alzheimer's Association; Icahn School of Medicine at Mount Sinai; VA Office of Research and Development	50-90	27	NCT00678431	Interventional	United States
Phase 1 (C)	Selenium	Assiut University	20-40	80	NCT03476044	Interventional	Egypt
Phase 1 (C)	Epigallocatechin-3-gallate (EGCG)	Parc de Salut Mar	14-29	87	NCT01699711	Interventional	Spain
Phase 0 (C)	Melatonin 5 mg; Sugar	Cairo University	20-60	30	NCT04959825	Interventional	Egypt

	Coated Tablets						
NA (W)	MCTprocal medical food; Milk/tricaprilin oil blend	Cerecin	18-55	0	NCT03635879	Interventional	United States
NA (W)	Wismemo	GenMont Biotech Incorporation; Chang Gung Memorial Hospital	55-95	0	NCT04094129	Interventional	Taiwan
NA (UN)	Ketogenic diet	Bristlecone Health, Inc.; University of Minnesota	18-80	30	NCT03859245	Interventional	United States
NA (UN)	Lithia water	American Society Of Thermalism And Climatology Inc	50-80	100	NCT02204969	Interventional	United States
NA (UN)	Supressi. T-Diet plus Range; High Protein.	Vegenat, S.A.	70 & older	184	NCT01192529	Interventional	Spain
NA (UN)	EGCG Lifestyle recommendations	Parc de Salut Mar; Fundacion IMIM; Barcelonabeta Brain Research Center, Pasqual Maragall Foundation	60-80	200	NCT03978052	Interventional	Spain
NA (UN)	Extra virgin olive oil	University of Bari Aldo Moro; Med & Food and Schena Foundation	50-70	24	NCT04229186	Interventional	Italy
NA (UN)	Magnesium sulfate	Metabolic Therapy Inc.	50 & older	100	NCT03038334	Interventional	United States
NA (UN)	PRJ212	Bioiberica	60-85	30	NCT02991235	Interventional	Spain
NA (UN)	Foods consistent with a MDP	University of East Anglia	55-74	108	NCT03673722	Interventional	United Kingdom
NA (UN)	Lipidic Blend	Team Foods Colombia S.A.; Biopolis S.L.	55-85	110	NCT02778581	Interventional	Spain
NA (UN)	Taurine	Third Military Medical University	18-80	200	NCT03410173	Interventional	China
NA (UN)	Turmeric, fisetin, green tea leaf extract, EPA, DHA and Vitamin D3	Cedars-Sinai Medical Center	55 & older	150	NCT02741804	Interventional	United States

NA (T)	lutein/zeaxanthin	Oregon Health and Science University; Oregon Partnership for Alzheimer's Research	55 & older	3	NCT00596024	Interventional	United States
NA (T)	Modified Atkins diet	Johns Hopkins University	12 to 65	20	NCT00952601	Interventional	United States
NA (T)	oral whey protein; Immunonutrition; Carbohydrate Drink	University Hospital Inselspital, Berne	75 & older	4	NCT03065348	Interventional	Switzerland
NA (R)	exogenous ketone salt (EKS) supplement	UniversitÃ© de Sherbrooke; Alzheimer's Association; NestlÃ© Health Science SA	55-80	56	NCT04466735	Interventional	Canada
NA (R)	Souvenaid	University of Miami; American Academy of Neurology	55-89	60	NCT04147624	Interventional	United States
NA (R)	Low protein diet	University of Genova	55-80	40	NCT05480358	Interventional	Italy
NA (R)	tricaprilin	Cerecin	18-65	80	NCT05028114	Interventional	Australia
NA (R)	low carbohydrate/ high fat diet; low fat/high carbohydrate diet	Wake Forest University Health Sciences; National Institute on Aging (NIA)	55-85	120	NCT03472664	Interventional	United States
NA (R)	HIGH and LOW meal ingestion	University of Washington	55 & older	80	NCT03070535	Interventional	United States
NA (R)	Multicultural Healthy Diet	Albert Einstein College of Medicine; Penn State University; Kaiser Permanente; University of Minnesota; National Institute on Aging (NIA)	40-65	326	NCT03240406	Interventional	United States
NA (R)	Citicoline supplement	Emory University	18& older	20	NCT05200208	Interventional	United States
NA (R)	Immunocal	Immunotec Inc.	55-85	25	NCT03448055	Interventional	Canada

NA (R)	lyophilized blueberry supplement	Duke University; U.S. Highbush Blueberry Council	55-85	12	NCT05172128	Interventional	United States
NA (R)	Heavy Cream	University of Washington; National Institute on Aging (NIA)	55& older	90	NCT04692441	Interventional	United States
NA (R)	Combination of High Protein Diet protocol	Massimo Venturelli, PhD; University Of Perugia; INCLIVA; Molde University College; University of Liverpool; Molecular Horizon S.r.l.; NestlÃ© Italiana S.p.A.; Universita di Verona	65-75	75	NCT05343611	Interventional	Italy
NA (R)	Transitional-state food therapeutic nutrition supplement	University of Oregon; Oregon Health and Science University; Oregon Partnership for Alzheimer's Research	18& older	50	NCT05007730	Interventional	United States
NA (R)	Juice Plus+	University of Alabama, Tuscaloosa; University of North Texas Health Science Center	55-90	150	NCT04656860	Interventional	United States
NA (R)	MIND Diet	Inha University Hospital	60-85	100	NCT05301868	Interventional	Korea, Republic of
NA (R)	MIND Diet	Inha University Hospital	60-79	300	NCT05023057	Interventional	Korea, Republic of
NA (NR)	Creatine Monohydrate	University of Kansas Medical Center; Alzheimer's Association	60-90	20	NCT05383833	Interventional	United States
NA (NR)	Betaquik MCT supplement	UniversitÃ© de Sherbrooke; NestlÃ©	60 & older	20	NCT04322461	Interventional	Canada
NA (C)	MMFS-205-SR	Neurocentria, Inc.; Ohio State University	55-85	10	NCT03531684	Interventional	United States
NA (C)	Copper	University Hospital, Saarland; University of Goettingen, Section Neurobiology (Head: Prof. Dr. T. Bayer), Germany	50-80	68	NCT00608946	Interventional	Germany
NA (C)	NT-020	University of South Florida	65-85	139	NCT01963767	Interventional	United States
NA (C)	NIC5-15	VA Office of Research and Development; National Center for Complementary and Integrative	Child, Adult,	15	NCT00470418	Interventional	United States

		Health (NCCIH); Humanetics Corporation	Older Adult				
NA (C)	NIC5-15	Humanetics Corporation; National Center for Complementary and Integrative Health (NCCIH); James J. Peters Veterans Affairs Medical Center	40-95	30	NCT01928420	Interventional	United States
NA (C)	Polyamine	Charite University, Berlin, Germany; Freie UniversitÃ¤t, Institute of Biology/Genetic, Berlin, Germany; Karl-Franzens-UniversitÃ¤t, Institute of Molecular Biosciences, Graz, Austria	60-90	100	NCT03094546	Interventional	Germany
NA (C)	Fermented Papaya Preparation; Granulated Sugar	University of Florida; Osato Research Institute	65-100	30	NCT02771366	Interventional	United States
NA (C)	secoisolariciresinol diglucoside	University of Saskatchewan; Saskatchewan Health Research Foundation	60-80	21	NCT01234506	Interventional	Canada
NA (C)	Niacin	McGill University Health Centre/Research Institute of the McGill University Health Centre; CIHR Canadian HIV Trials Network	18& older	16	NCT02018965	Interventional	Canada
NA (C)	Nutritional Supplement	National Institute on Aging (NIA); Burke Medical Research Institute	50 & older	40	NCT00013923	Interventional	United States
NA (C)	Novasoy	University of Wisconsin, Madison; National Institutes of Health (NIH); National Institute on Aging (NIA)	55 & older	72	NCT00205179	Interventional	United States
NA (C)	Mito-Food Plan; Cellular Repair Therapy	Perseverance Research Center, LLC; Cerulean Advanced Fitness and Wellness	55-90	5	NCT03630419	Interventional	United States
NA (C)	Fish Oil; Lipoic Acid; Fish Oil; Lipoic Acid	Oregon Health and Science University; National Institute on Aging (NIA); National Center for Research Resources (NCRR)	55 & older	39	NCT00090402	Interventional	United States

NA (C)	NutriterraTM	Nuseed Americas Inc.; Nutrasource Pharmaceutical and Nutraceutical Services, Inc.	18-80	132	NCT03937206	Interventional	Canada
NA (C)	Elderberry Juice	University of Missouri-Columbia	50 & older	24	NCT02414607	Interventional	United States
NA (C)	grape seed polyphenolic extract, resveratrol	Johns Hopkins University; Icahn School of Medicine at Mount Sinai	50-90	14	NCT02502253	Interventional	United States
NA (C)	Etanercept; Curcum.Luteol.Theaflav.Lip.Acid,FishOil,Quercet.,Resveratr.	Life Extension Foundation Inc.	60-85	12	NCT01716637	Interventional	United States
NA (C)	Scelectium Tortuosum	Woodbury, Michel, M.D.; PL Thomas & Co., Inc.	45-65	20	NCT01805518	Interventional	Puerto Rico
NA (C)	tricaprilin; standard meal; high-fat meal	Cerecin	18-55	20	NCT03551769	Interventional	Australia
NA (C)	Low-Carbohydrate Diet; Low-Fat Diet	Wake Forest University Health Sciences	50-85	25	NCT02984540	Interventional	United States
NA (C)	diet high in saturated fat (SF); glycemic index (GI), and salt (Na+) to a diet low in these nutritional parameters	Wake Forest University Health Sciences	45-65	60	NCT02463084	Interventional	United States
NA (C)	Modified Atkins Diet; NIA Diet for Seniors	Johns Hopkins University	60 & older	38	NCT02521818	Interventional	United States
NA (C)	MCT oil	University of Alberta	50-90	20	NCT04396015	Interventional	Canada

NA (C)	ReBuilder	Genescient Corporation	Child, Adult, Older Adult	50	NCT03611439	Interventional	United States
NA (C)	High phenolic EVOO intake; No High phenolic EVOO intake	University of Peloponnese; National and Kapodistrian University of Athens; Harokopio University	54-84	21	NCT04215367	Interventional	Greece
NA (C)	Lipid drink	University of Washington; National Institute on Aging (NIA)	55 & older	30	NCT05218018	Interventional	United States
NA (C)	Extra virgin olive oil	Auburn University	55-75	25	NCT03824197	Interventional	United States
NA (C)	pomace oil	National Research Council, Spain; Hospitales Universitarios Virgen del RocÃo	18-26	20	NCT04559828	Interventional	Spain
NA (C)	Caloric restriction	Iowa State University; National Institute on Aging (NIA)	70-85	96	NCT03872375	Interventional	United States
NA (C)	1-month of 60-40 MCT oil; 1-month of C8 MCT oil	UniversitÃ© de Sherbrooke; Fondation Vitae	65 & older	20	NCT02709356	Interventional	Canada
NA (C)	Genistein	FundaciÃ³n para la InvestigaciÃ³n del Hospital ClÃnico de Valencia; University of Valencia	18& older	27	NCT01982578	Interventional	Spain
NA (C)	cycloastragenol	Chippewa Valley Eye Clinic; TA-Sciences	50-70	48	NCT02530255	Interventional	United States
NA (C)	ginsenoside, green tea polyphenols and marine collagen peptide	XuanwuH 2; Xuanwu Hospital, Beijing	50-79	60	NCT04279418	Interventional	China
NA (C)	ORGONO Living Silica Acacia Gum-MMST Powder	FundaciÃ³ Eurecat; Silicium Laboratories S.L.; San Antonio Technologies S.L.; Catholic University of Murcia (UCAM); Zaidin Experimental Station, Spanish Council for Scientific Research (CSIC) Agency	40-65	47	NCT05116982	Interventional	Spain

NA (C)	Oxaloacetate (OAA)	Russell Swerdlow, MD; University of Kansas Medical Center	18 & older	6	NCT02063308	Interventional	United States
NA (C)	EPA; DHA	Taichung Veterans General Hospital	65-105	163	NCT04972643	Interventional	Taiwan
NA (C)	Erinacine A-enriched Hericium Erinaceus Mycelia	Chung Shan Medical University; Grape King Bio Ltd.	50-90	68	NCT04065061	Interventional	Taiwan
NA (C)	Magtein	Stanford University; Magceutics, Inc.	60 & older	17	NCT02210286	Interventional	United States
NA (C)	Vitis vinifera extract	Azienda Ospedaliera Universitaria Policlinico "G. Martino"	55-75	111	NCT03145987	Interventional	
NA (C)	Probiotic supplemented intervention	XuanwuH 2; First Hospital of Tsinghua University; Beijing Normal University; Xuanwu Hospital, Beijing	55 & older	90	NCT03991195	Interventional	China
NA (C)	Caprylic Triglyceride	University of Miami; National Multiple Sclerosis Society; Cerecin	18-59	124	NCT01848327	Interventional	United States
NA (C)	MCT oil	UniversitÃ© de Sherbrooke	18 & older	10	NCT02409927	Interventional	
NA (C)	Tart Cherry Juice	Kronos Longevity Research Institute; Vanderbilt University	55-80	12	NCT00847743	Interventional	United States
NA (C)	Early Harvest Extra Virgin Olive Oil	Aristotle University Of Thessaloniki; Greek Alzheimer's Association and Related Disorders; Ellis-Farm, Eliama Daily Value Gold (ellis-farm.com)	18-65	30	NCT04120675	Interventional	Greece
NA (C)	MIND Diet	Practitioners Alliance Network	Child, Adult, Older Adult	25	NCT02260167	Interventional	United States
NA (C)	Tahini	Harokopio University	20-40	20	NCT04608747	Interventional	Greece
NA (C)	NeuroQ	LifeSeasons Inc.; KGK Science Inc.	45 & older	40	NCT04149639	Interventional	United States
NA (A, NR)	Meganatural-Az Grapeseed Extract	Hillel Grossman; National Center for Complementary and Integrative Health (NCCIH); Icahn School of Medicine at Mount Sinai	Child, Adult, Older Adult	20	NCT02033941	Interventional	United States

(UN)	brain polypeptide solution; same package of	Peking Union Medical College Hospital; Zhitong Biopharma CO.,LTD	50-85	200	NCT03978338	Interventional	
(UN)	Compare concentration of various vitamins and biomarkers in mild cognitive impairment (MCI) or mild dementia patient.	Oslo University Hospital; South Eastern Area Health Service	Child, Adult, Older Adult	180	NCT01479855	Observational	Norway
(R)	Whole egg intake and choline concentration in aging brain	University of Kansas Medical Center; Egg Nutrition Center	60-85	60	NCT05021211	Observational	United States
(C)	CerefolinNAC Â®	Pamlab, Inc.; InfoMedics, Inc.	50-80	204	NCT01370954	Observational	United States
(C)	alphatocopherol; vitamin E; replacement for Selenium	Frederick Schmitt; National Institute on Aging (NIA); National Cancer Institute (NCI); University of Kentucky	60-90	4246	NCT00040378	Observational	Puerto Rico
(C)	Cocoa supplement and vitamins	Wake Forest University Health Sciences; National Institute on Aging (NIA); Brigham and Women's Hospital	65 & older	2262	NCT03035201	Observational	United States

C= Completed; NR= Non-recruiting; A, NR= Active non-recruiting; EI: Enrolling by invitation; T: Terminated; UN: Unknown; NA: Not applicable

Table S 4. Clinical trial data of dietary interventions associated with diseases like Amyotrophic lateral sclerosis, Huntington disease and Spinal muscular atrophy.

Phase & Status	Intervention	Sponsor	Age	Enrollment	Location	NCT Number	Condition
Phase 3 (T)	KetoCal	Johns Hopkins University;Nutricia North America;Cornell University	18 years & older	1	United States	NCT01016522	Amyotrophic lateral sclerosis
Phase 3 (C)	Vitamin E	Lawson Health Research Institute	18 years & older	32	Canada	NCT00372879	Amyotrophic lateral sclerosis
Phase 2 (C)	Oxepa; Jevity 1.5; Jevity 1.0	Massachusetts General Hospital;Muscular Dystrophy Association	18 years & older	28	United States	NCT00983983	Amyotrophic lateral sclerosis
Phase 2 (UN)	Tocotrienols	University of Malaya	Child, Adult, Older Adult	20	Malaysia	NCT04140136	Amyotrophic lateral sclerosis
Phase 2 (R)	Liposomed polyphenols resveratrol and curcumin;Isocaloric Diet	FundaciÃ³n Universidad CatÃ³lica de Valencia San Vicente MÃ¡rtir	18-75	60	Spain	NCT04654689	Amyotrophic lateral sclerosis
Phase 2 (R)	L-Serine	Elijah W. Stommel;Brain Chemistry Labs, Institute for Ethnomedicine;Dartmouth-Hitchcock Medical Center	18 years & older	50	United States	NCT03580616	Amyotrophic lateral sclerosis
Phase 2 (C)	creatine monohydrate	National Center for Complementary and Integrative Health (NCCIH);Office of Dietary Supplements (ODS)	21-80	110	United States	NCT00070993	Amyotrophic lateral sclerosis
Phase 2 (C)	Albumin	Instituto Grifols, S.A.;Grifols Biologicals, LLC	18-70	13	Spain	NCT02479802	Amyotrophic lateral sclerosis
Phase 1 (C)	high-caloric fatty diet	Albert Christian Ludolph, Prof.; Nutritia GmbH, 91052 Erlangen, Germany; University of Ulm	18 years & older	64	Germany	NCT04172792	Amyotrophic lateral sclerosis
Phase 1 (C)	PolyMVA	University of Missouri-Columbia;Band of Hope Foundation	Child, Adult,	12	United States	NCT04557410	Amyotrophic lateral sclerosis

			Older Adult				
Phase 1 (W)	Spirit1	Herb Spirit;Carmel Medical Center	18-75	0	Israel	NCT02588807	Amyotrophic lateral sclerosis
NA (W)	Modified Paleolithic diet	Terry L. Wahls;Muscular Dystrophy Association;University of Iowa	18-80	0		NCT03659422	Amyotrophic lateral sclerosis
NA (C)	Calogen	University of Ulm	18 years & older	207	Germany	NCT02306590	Amyotrophic lateral sclerosis
NA (NR)	Beta Hydroxybutyrate Ester	University of Ulm	18 years & older	76	Germany	NCT04820478	Amyotrophic lateral sclerosis
NA (C)	Oral nutritional supplementation	University Hospital, Limoges;Laboratoires NUTRICIA	18 years & older	229	France	NCT02152449	Amyotrophic lateral sclerosis
NA (C)	Probiotic	Avera McKennan Hospital & University Health Center	18 years & older	5	United States	NCT03324399	Amyotrophic lateral sclerosis
NA (R)	Hirsutella Sinensis Nutrient Supplements	Chang Gung Memorial Hospital;Grape King Bio Ltd.	50and older	100	Taiwan	NCT05284149	Amyotrophic lateral sclerosis
(C)	PUFA	University of Jena;Jena University Hospital	18-90	50	Germany	NCT02572479	Amyotrophic lateral sclerosis
(C)	AARP Diet and health	National Institute of Environmental Health Sciences (NIEHS);National Institutes of Health Clinical Center (CC)	50-80	21	United States	NCT00377351	Amyotrophic lateral sclerosis
Phase 3 (C)	Tagatose; Sugar Substitute Splenda	Robert Lodder;University of Kentucky;Spherix Incorporated	18-75	494	India	NCT00955747	Huntington
Phase 3 (C)	Calcium salts; cholecalciferol; zoledronic acid	Alliance for Clinical Trials in Oncology;National Cancer Institute (NCI)	40and older	439	United States	NCT00022087	Huntington
Phase 3 (A, NR)	Nitazoxanide; Vitamin Super B-Complex	Romark Laboratories L.C.	18-120	1407	United States	NCT04359680	Huntington
Phase 3 (T)	Creatine Monohydrate	Massachusetts General Hospital;University of	18 years & older	553	New Zealand	NCT00712426	Huntington

		Rochester;National Center for Complementary and Integrative Health (NCCIH)					
Phase 3 (C)	calcium gluconate;magnesium sulfate;oxaliplatin	Alliance for Clinical Trials in Oncology;National Cancer Institute (NCI)	18 years & older	362	Puerto Rico	NCT01099449	Huntington
Phase 2 (T)	Omega 3 Fatty Acid	Marshall University;Edwards Foundation, Inc.	18 years & older	16	United States	NCT00899353	Huntington
Phase 1 (C)	ubiquinol	University of Rochester;Kaneka Corporation	18 years & older	6	United States	NCT00980694	Huntington
NA (R)	Melatonin	The University of Texas Health Science Center, Houston;Huntington's Disease Society of America	18-75	20	United States	NCT04421339	Huntington
NA (C)	Vitamin D3	Southwest Oncology Group;National Cancer Institute (NCI)	18-50	208	United States	NCT01097278	Huntington
NA (C)	Amino Acid formula	NestlÃ©	2 Months to 12	37	United States	NCT01569776	Huntington
NA (C)	Extensively hydrolyzed, whey protein infant formula; Extensively hydrolyzed, casein protein infant formula	NestlÃ©	2 Months to 12		United States	NCT01278446	Huntington
NA (C)	Three sachet of protein supplement to be mixed in with water used daily	Shahid Beheshti University	18-65	80	Islamic Republic of	NCT04300517	Spinal muscular atrophy
NA(R)	Hirsutella Sinensis Nutrient Supplements	Chang Gung Memorial Hospital;Grape King Bio Ltd.	50 and older	100	Taiwan	NCT05284149	Spinal muscular atrophy

C= Completed; NR= Non-recruiting; W: Withdrawn; A, NR= Active non-recruiting; EI: Enrolling by invitation; T: Terminated; UN: Unknown; NA: Not applicable
